# Chemical variability for authenticated and commercial *Artemisia absinthium* L. essential oils with thujones on tephritid fruit flies: Mediterranean fruit fly *Ceratitis capitata* (Wiedemann) and Caribbean fruit fly *Anastrepha suspensa* (Loew)

**DOI:** 10.3389/fpls.2025.1674428

**Published:** 2025-11-20

**Authors:** Nurhayat Tabanca, Kevin R. Cloonan, Xiangbing Yang, Ayse Baldemir Kılıc, Betul Demirci

**Affiliations:** 1 Subtropical Horticulture Research Station, United States Department of Agriculture, Agricultural Research Service, Miami, FL, United States; 2 Department of Pharmaceutical Botany, Faculty of Gulhane Pharmacy, University of Health Sciences, Ankara, Türkiye; 3 Department of Pharmacognosy, Faculty of Pharmacy, Anadolu University, Eskisehir, Türkiye

**Keywords:** semiochemical, *Artemisia absinthium*, wormwood, α-thujone, β-thujone, medfly, caribfly, integrated pest management

## Abstract

**Introduction:**

Fruit flies, belonging to the family Tephritidae, such as the Mediterranean fruit fly (*Ceratitis capitata* Wiedemann) and the Caribbean fruit fly (*Anastrepha suspensa* Loew), are recognized as major agricultural pests worldwide. Their larval stages infest a wide array of fruits and vegetables, causing significant economic losses through direct damage to crops and restrictions on international trade. Conventional pest management, heavily reliant on synthetic pesticides, has led to health concerns and the emergence of pesticide resistance. In response, semiochemicals, particularly essential oils and their constituents, are emerging as promising alternatives.

**Methods:**

In this study, authenticated *Artemisia absinthium* L. (wormwood, Asteraceae) (A_sd_) essential oil (EO) was compared with five lab-distilled commercial *A. absinthium* EOs (A_1_ to A_5_) using gas chromatography with a flame ionization detector (GC–FID) and gas chromatography–mass spectrometry (GC–MS), and essential oils were tested in behavioral assays on sterile male *C. capitata*. Key components were evaluated for their potential attraction of male *C. capitata*, and the toxicities of compounds to female *A. suspensa* were determined.

**Results:**

Cluster analysis revealed three major groups of compounds: β-thujone and (*Z*)-β-ocimene epoxide, β-thujone and camphor, and only camphor-rich. In short-range attraction bioassays, A_sd_ and A_1_ samples captured the most male *C. capitata* at 30 minutes. These findings were linked to a higher α,β-thujone content in samples A_sd_ (41.04%) and A1 (29.6%). A set of bioassays were conducted to compare the response of *C. capitata* to α-thujone, α,β-thujone, and tea tree oil, a strong medfly attractant. Medflies were similarly attracted to both α-thujone and α,β-thujone from 30 to 90 minutes. In a subsequent bioassay, α-thujone and α,β-thujone demonstrated strong toxicity to adult female *A. suspensa*, with the LD_50_ values being 0.21 and 0.14 μg/μL, respectively.

**Discussion:**

These findings demonstrate that thujones have both attractant properties for male *C. capitata* and significant toxicity to *A. suspensa*, making them promising candidate compounds for integration into comprehensive integrated pest management strategies.

## 1 Introduction

Tephritid fruit flies are economically important insect pests of fruit commodities that threaten tropical, subtropical, and Mediterranean regions. They are considered highly polyphagous and potential exotic invaders (Fimiani, 1989; Vera et al., 2002; Puche et al., 2005; Suckling et al., 2008; Vargas et al., 2010; Navarro-Campos et al., 2011; Aluja et al., 2014; Epsky et al., 2014; Papadopoulos, 2014; Qin et al., 2015; Suckling et al., 2016; Weldon et al., 2018; Dumont and Oliva, 2024; Zhao et al., 2024). Among the invaders, Mediterranean fruit fly (medfly), *Ceratitis capitata* Wiedemann, Mexican fruit fly (mexfly), *Anastrepha ludens* Loew, and Oriental fruit fly, *Bactrocera dorsalis* Hendel, are of major quarantine importance to the United States (Froerer et al., 2010; Mangan et al., 2011; Steck et al., 2019; Duyck et al., 2022; Salas et al., 2023; Davydova et al., 2023; Shelly et al., 2023; Roh et al., 2023). Florida faces a high risk from invasive insect pests. This is due to several factors—its unique geology, favorable ecological conditions, numerous international ports of entry, and increasing tourism—all of which make the state particularly vulnerable and alter the ecosystem (Frank and McCoy, 1995; Brown et al., 2006; Wagner and Van Driesche, 2010; Pinkerton et al., 2019; Inserra et al., 2023; Raparelli et al., 2025).

Florida has sporadically faced numerous incursions of *C. capitata*; however, due to strict control strategies and successful eradication programs implemented by regulatory agencies, the medfly has not become an established pest in Florida (Simanton, 1958; Cunningham, 1989; Pinero et al., 2014; Tabanca et al., 2019; Skelley et al., 2025). However, the Caribbean fruit fly (caribfly), *Anastrepha suspensa* Loew, is established in Florida, where it is a production pest of guava and a quarantine pest of citrus. To ensure that citrus crops remain free of this pest, they must be grown in areas certified under the “Fly-Free Zone Certification Protocol” (Gould, 1998). These zones must be clear of host plants, such as loquat, *Eriobotrya japonica* Lindl., rose apple, *Syzygium jambos* (L.) Alst., Cattley guava, *Psidium cattleianum* Sabine, common guava, *Psidium guajava* L., and Surinam cherry, *Eugenia uniflora* L (Nguyen et al., 1992; Simpson, 1993; Gould, 1998; Weems et al., 2001; Heve et al., 2017). Fruit fly management relies on monitoring through a network of lure-baited traps and insecticide applications; however, continuous use of insecticides raises concerns of resistance development, environmental contamination, and negative impacts on non-target organisms (Troetschler, 1983; Wang and Messing, 2006; Shelly et al., 2012; Hsu et al., 2011; Shikano et al., 2022; Antonio et al., 2023; Hoskins et al., 2023). Therefore, there is an urgent need to develop biorational alternatives for managing tephritid fruit flies. One such biorational management tool is the sterile insect technique (SIT) for controlling and eradicating *C. capitata* (Juan-Blasco et al., 2013; Manrakhan and Addison, 2014; Pimentel et al., 2017; Mason, 2024). In theory, sterile males are released and mate with wild females, thereby preventing reproduction and leading to population suppression. The success of SIT requires sterile males to compete with wild males (Suckling et al., 2011; Diouf et al., 2024; Li et al., 2024).

Semiochemicals, defined as chemical signals involved in inter- or intra-specific insect communication, are constantly investigated and utilized as biorational tools for pest management. Their primary applications involve either attracting male and female fruit flies into traps for monitoring or mass capture or promoting repellency to protect exposed fruits from infestation. Furthermore, semiochemicals play a role in male lek formation (grouping to attract females), calling males within these aggregations, and releasing pheromones to draw in females by improving the competitiveness of sterile males (Vanickova et al., 2012; Shelly, 2018; Tejada et al., 2020; Dumont and Oliva, 2024). Male-specific semiochemicals are also used in traps for monitoring *C. capitata*. For instance, the synthetic male-specific attractant trimedlure [*tert*-butyl 4 (and 5)-chloro-2-methylcyclohexane-1-carboxylate] is a key component in traps targeting male *C. capitata* (Cunningham, 1989; Shelly et al., 1996; Tan et al., 2014; Shelly and Cloonan, 2023). Despite its effectiveness, trimedlure is produced by only one manufacturer globally. This limited supply highlights the need to develop alternative, plant-based male-specific attractants for *C. capitata*. The success of SIT programs is directly linked to the quality and competitiveness of the released sterile males. The use of volatile attractants is crucial to ensure that they exhibit mating behavior similar to their wild counterparts and effectively compete for mates.

Essential oils (EOs), rich in diverse bioactive compounds, are considered a prime resource of male-specific kairomones for *C. capitata*. For example, a sesquiterpene hydrocarbon, (+)-α-copaene, found in *Angelica archangelica* L. EO, was identified as highly attractive to medfly males, and the oil was used in monitoring traps during early eradication programs in Florida before the identification of trimedlure (Steiner et al., 1957; Simanton, 1958; Segura et al., 2018). While research efforts testing the attractiveness of (+)-α-copaene have been successful and found that it is three times more attractive to male *C. capitata* than trimedlure, it has not been widely used in the field. This is because isolating and synthesizing it is expensive, and its availability from natural sources is limited (Flath et al., 1994a, b; Shelly et al., 2023; Lull et al., 2023; Koening et al., 1994). Additional studies have shown that α-copaene analogs, such as α-ylangene and β-copaene, demonstrated short-range attractiveness to *C. capitata* males but were not strong attractants in the field (Flath et al., 1994a, b). Accordingly, research efforts were devoted to developing alternative natural sources of medfly attractants. Ginger root oil, *Zingiber officinale* Roscoe, another non-host plant, also increased the mating competitiveness of males, and the effect was long-lasting (Shelly and Mclnnis, 2001). Searching for new natural sources of α-copaene, volatile chemicals from avocado (*Persea americana* Mill.), litchi (*Litchi chinensis* Sonn.), and ficus (*Ficus benjamina* L.) wood were investigated. Behavior and electroantennogram responses to male *C. capitata* showed that litchi elicited the highest responses and ficus the lowest, and the attraction to avocado genotypes varied. This study did not directly correlate medfly attraction with α-copaene levels, suggesting that other sesquiterpenes may influence male responses (Niogret et al., 2011). Following the attraction of medfly males to ginger root EO, short-range bioassays demonstrated that linalool was primarily responsible for the attraction to ginger root oil (Niogret et al., 2017). In studies of the short-range attraction of sterile *C. capitata*, we found that *Melaleuca alternifolia* (Maiden & Betche) Cheel, *Tetradenia riparia* (Hochst.) Codd, and *Juniperus foetidissima* Willd EOs were highly attractive (Shelly and Epsky, 2015; Tabanca et al., 2020; Blythe et al, 2020; Kurtca et al., 2021).

In our ongoing search for new monitoring and control tools of *C. capitata* and other tephritid fruit flies, we explored the EOs of *Artemisia absinthium* L. (Asteraceae) as a potential source of kairomones. *A. absinthium*, commonly known as wormwood, is a strong aromatic plant native mainly to Europe, but represented in the Mediterranean region and Asia (Mihajilov-Krstev et al., 2014; Nemeth, 2016). Both EO and wormwood extracts have been widely used in many gastric herbal preparations, dietary supplements, and alcoholic beverages, and for agricultural purposes, such as acaricidal, antifungal, and insecticidal properties and allelopathic effects (Lachenmeier and Uebelacker, 2010; Abad et al., 2012; Umpierrez et al., 2012; Llorens-Molina and Vacas, 2015). Its EO is connected with its potential natural insecticide primarily due to the presence of thujone isomers (Abad et al., 2012; Mihajilov-Krstev et al., 2014; Nguyen and Nemeth, 2016). Thujones are found in various EOs, including *A. absinthium*, with their ratio varying depending on the plant source and developmental stage (Nemeth, 2016).

The current study aimed to i) analyze the chemical composition of authenticated *A. absinthium* EO and compare it with lab-distilled *A. absinthium* EOs purchased as plant tissues from various local herbal supplies in Turkey using gas chromatography–flame ionization detection (GC–FID) and gas chromatography–mass spectrometry (GC–MS), ii) evaluate *A. absinthium* EOs in behavioral assays with sterile male *C. capitata*, and iii) evaluate the key compounds in the behavioral assays for *C. capitata* and determine their toxicity against female Caribbean fruit flies, *A. suspensa*.

## 2 Materials and methods

### 2.1 Materials

**Figure 1:**
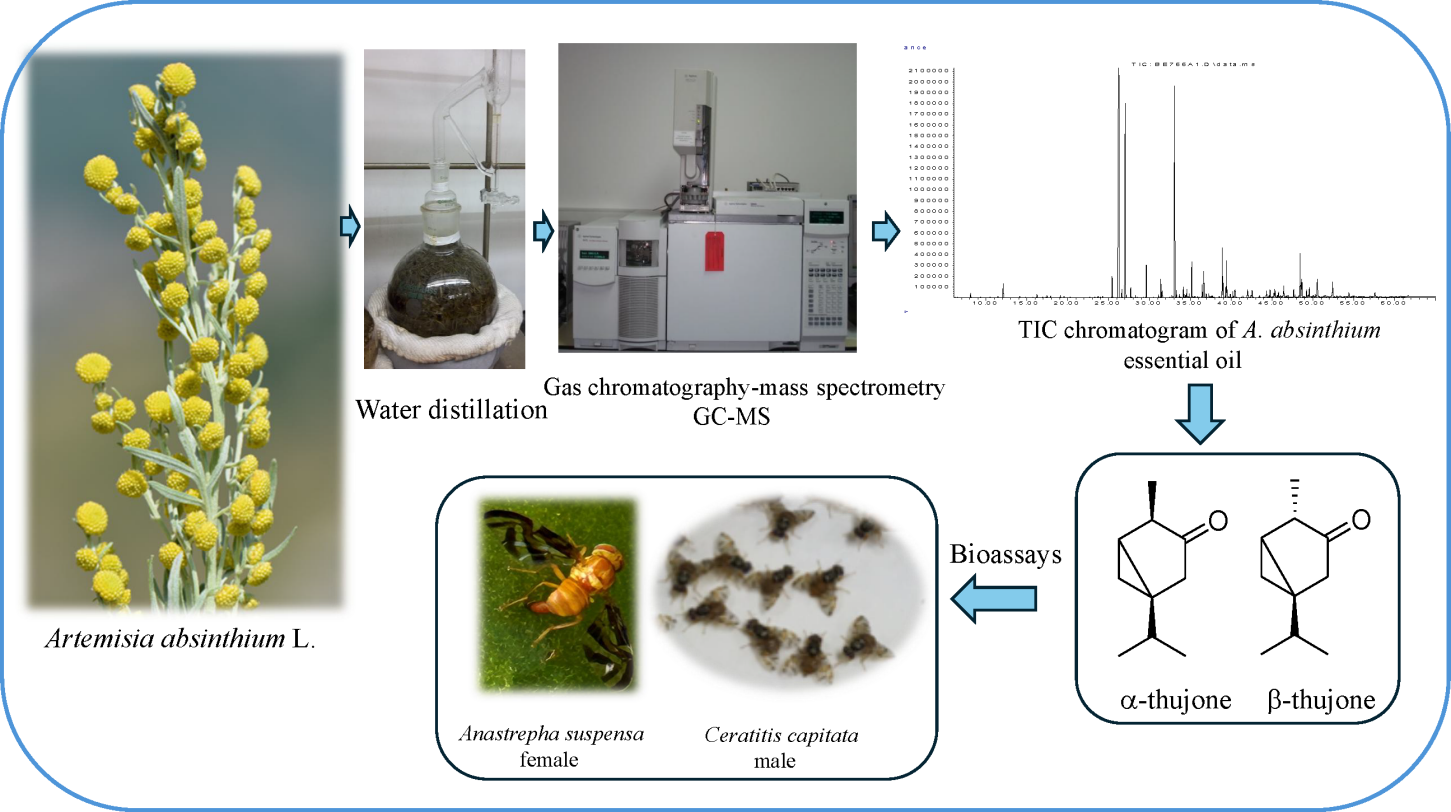
Schematic representation of the methodology used to analyze *Artemisia absinthium* EOs by GC–MS system and test the key compounds in the behavioral and toxicity assays for *Ceratitis capitata* and *Anastrepha suspensa*. EOs, essential oils; GC–MS, gas chromatography–mass spectrometry.

*A. absinthium* used as an authenticated/standard (A_sd_) was obtained from the natural sources of Istanbul, Zeytinburnu Medicinal Plants Garden in Turkey. Commercial samples A_1_ to A_5_ were purchased from Turkish herbal shops as loose plant parts per sample. The *A. absinthium* aerial parts were subjected to hydrodistillation for 3 hours using a Clevenger-type apparatus to obtain essential oils. The percentage yields were calculated on a dry weight basis as given in **Supplementary Table S1** (**Supplementary Material**). The oils were dried over anhydrous sodium sulfate to remove moisture and stored at +4°C until analysis and tested further. α-Thujone (CAS No. 546-80-5) and α,β-thujone mixture (Product No. PHL82669) were purchased from Sigma-Aldrich Inc. (St. Louis, MO, USA). A schematic representation of the methodology is given in **Figure 1**.

### 2.2 Analysis of essential oils

#### 2.2.1 Gas chromatography–mass spectrometry analysis

The GC–MS analysis was carried out with an Agilent 5975 GC-MSD system (Santa Clara, CA, USA). Innowax FSC column (60 m × 0.25 mm, 0.25 µm film thickness) was used with helium as carrier gas (0.8 mL/min). GC oven temperature was kept at 60°C for 10 minutes, programmed to 220°C at a rate of 4°C/min, kept constant at 220°C for 10 minutes, and then programmed to 240°C at a rate of 1°C/min. The split ratio was adjusted to 40:1. The injector temperature was set to 250°C. Mass spectra were recorded at 70 eV. Mass range was from *m/z* 35 to 450.

#### 2.2.2 Gas chromatography with flame ionization detector analysis

The GC analysis was carried out using an Agilent 6890N GC system. The FID detector temperature was 300°C. To obtain the same elution order using GC–MS, simultaneous auto-injection was conducted on a duplicate of the same column, applying the same operational conditions. Relative percentage amounts of the separated compounds were calculated from FID chromatograms. The analysis results are given in **Table 1**.

**Table 1:** The chemical composition of essential oils of *Artemisia absinthium*.

#	RRI ^a^	Compound	A_sd_ %^b^	A_1_ % ^b^	A_2_ % ^b^	A_3_ % ^b^	A_4_ % ^b^	A_5_ % ^b^	IM^c^
1	1014	Tricyclene	–	–	0.1	0.1	0.2	0.1	*t*_R_, MS
2	1032	α-Pinene	0.3 ± 0	0.9	0.6	0.9	0.7	1.0	*t*_R_, MS
3	1035	α-Thujene	–	0.1	<0.1	<0.1	<0.1	0.1	*t*_R_, MS
4	1076	Camphene	–	1.0	2.1	2.4	2.9	3.2	*t*_R_, MS
5	1093	Hexanal	–	–	0.1	0.1	0.2	–	*t*_R_, MS
6	1118	β-Pinene	0.1 ± 0	2.2	–	–	–	–	*t*_R_, MS
7	1132	Sabinene	0.93 ± 0.05	0.2	–	–	–	–	*t*_R_, MS
8	1174	Myrcene	0.1 ± 0	0.2	–	–	–	0.2	*t*_R_, MS
9	1185	Isobutyl 2-methyl butyrate	–	0.1	–	–	–	–	MS
10	1188	α-Terpinene	0.1 ± 0	0.5	0.4	0.4	0.3	0.5	*t*_R_, MS
11	1203	Limonene	–	0.5	0.3	0.2	–	0.4	*t*_R_, MS
12	1203	2-Methyl butyl isobutyrate	–	0.6	–	–	–	–	MS
13	1213	18-Cineole	0.1 ± 0	0.5	0.1	0.2	0.2	0.1	*t*_R_, MS
14	1225	(*Z*)-3-Hexenal	0.23 ± 0.05	–	–	–	–	–	MS
15	1241	Butyl-2-methylbutyrate	–	0.1	–	–	–	–	MS
16	1246	(*Z*)-β-Ocimene	0.2 ± 0	–	–	–	–	–	MS
17	1255	γ-Terpinene	0.1 ± 0	1.0	0.8	0.7	0.6	0.9	*t*_R_, MS
18	1280	*p*-Cymene	0.2 ± 0	3.9	2.1	2.4	2.5	2.4	*t*_R_, MS
19	1299	2-Methylbutyl isovalerate	–	2.2	1.5	0.7	1.2	1.0	MS
20	1348	6-Methyl-5-hepten-2-one	–	0.1	0.1	0.1	–	0.2	MS
21	1437	α-Thujone	1.17 ± 0.05	1.2	–	–	–	–	*t*_R_, MS
22	1450	*trans*-Linalool oxide (*Furanoid*)	–	–	<0.1	–	–	–	MS
23	1451	β-Thujone	39.87 ± 0.49	28.4	–	1.4	1.0	0.2	*t*_R_, MS
24	1474	*trans*-Sabinene hydrate	–	0.3	–	–	–	–	MS
25	1476	(*Z*)-β-Ocimene epoxide	17.87 ± 0.37	–	–	–	–	–	MS
26	1478	*cis*-Linalool oxide (*Furanoid*)	–	0.1	–	–	–	–	MS
27	1482	(*Z*)-3-Hexenyl-2-methyl butyrate	–	0.1	0.1	–	–	0.1	MS
28	1497	α-Copaene	–	0.3	–	0.2	–	0.4	MS
29	1498	(*E*)-β-Ocimene epoxide	0.4 ± 0.14	–	–	–	0.6	–	MS
30	1532	Camphor	–	30.5	40.1	50.8	45.9	42.1	*t*_R_, MS
31	1553	Linalool	1.67 ± 0.05	0.7	1.8	0.9	1.0	1.7	*t*_R_, MS
32	1556	*cis*-Sabinene hydrate	–	0.2	–	–	–	0.2	MS
33	1571	*trans*-*p*-Menth-2-en-1-ol	–	0.3	0.1	0.3	–	0.3	MS
34	1611	Terpinen-4-ol	0.4 ± 0	2.8	2.2	2.1	1.2	2.1	*t*_R_, MS
35	1612	β-Caryophyllene	0.93 ± 0.05	0.4	0.6	0.4	0.4	0.2	*t*_R_, MS
36	1614	Rose furan epoxide	0.1 ± 0	–	–	–	–	–	MS
37	1638	*cis*-*p*-Menth-2-en-1-ol	–	0.2	–	0.2	–	0.2	MS
38	1648	Myrtenal	–	0.3	–	–	–	–	MS
39	1658	Sabinyl acetate	13.87 ± 0.12	–	–	–	0.3	–	MS
40	1663	*cis*-Verbenol	–	0.1	–	–	–	–	*t*_R_, MS
41	1669	Sesquisabinene	0.2 ± 0	–	–	–	–	–	MS
42	1670	*trans*-Pinocarveol	–	0.3	–	–	–	–	*t*_R_, MS
43	1683	*trans*-Verbenol	–	0.3	0.3	0.3	0.3	0.4	*t*_R_, MS
44	1687	α-Humulene	0.27 ± 0	–	–	–	–	–	*t*_R_, MS
45	1688	Selina-4,11-diene (=*4,11-Eudesmadiene*)	0.2 ± 0	–	–	–	–	–	MS
46	1697	Carvotanacetone	–	0.8	–	–	–	–	MS
47	1704	γ-Curcumene	0.47 ± 0.05	–	–	–	–	–	MS
48	1705	Fragranyl acetate	0.33 ± 0.09	–	–	–	–	–	*t*_R_, MS
49	1706	α-Terpineol	0.17 ± 0.05	0.3	0.3	0.5	0.2	0.3	*t*_R_, MS
50	1719	Borneol	–	0.8	0.4	0.6	0.4	0.7	*t*_R_, MS
51	1725	Verbenone	–	0.1	0.1	–	–	–	*t*_R_, MS
52	1726	Germacrene D	0.27 ± 0.05	1.3	0.4	0.1	0.1	0.1	*t*_R_, MS
53	1737	Carvenone	–	0.3	–	–	–	–	*t*_R_, MS
54	1742	β-Selinene	1.8 ± 0	–	0.7	0.2	0.8	0.7	MS
55	1751	Carvone	–	0.5	–	0.3	–	–	*t*_R_, MS
56	1755	Bicyclogermacrene	–	0.2	–	–	–	–	MS
57	1758	*cis*-Piperitol	–	0.1	–	0.1	–	–	*t*_R_, MS
58	1773	δ-Cadinene	–	0.2	–	–	–	–	MS
59	1786	*ar*-Curcumene	0.5 ± 0	–	–	–	–	–	MS
60	1786	Neryl propionate	1.1 ± 0	–	–	–	–	–	MS
61	1797	*p*-Methyl acetophenone	–	0.4	0.4	0.4	0.5	0.2	*t*_R_, MS
62	1802	Cumin aldehyde	–	0.3	0.1	0.4	0.3	0.1	*t*_R_, MS
63	1804	Myrtenol	–	0.1	–	–	–	–	MS
64	1807	Perilla aldehyde	–	0.1	–	0.4	0.5	1.0	MS
65	1808	Nerol	0.27 ± 0.05	–	–	–	–	–	*t*_R_, MS
66	1827	(*E*,*E*)-2,4-Decadienal	–	–	–	0.1	0.5	–	MS
67	1845	(*E*)-Anethol	–	0.1	0.1	0.2	0.6	–	*t*_R_, MS
68	1845	*trans*-Carveol	–	–	–	–	–	–	*t*_R_, MS
69	1857	Geraniol	<0.1	0.1	–	–	–	–	*t*_R_, MS
70	1863	Fragranyl 2-methyl butyrate	0.7 ± 0	–	–	–	–	–	MS
71	1864	*p*-Cymen-8-ol	–	0.4	0.2	0.2	–	–	*t*_R_, MS
72	1871	Neryl isovalerate	1.67 ± 0.05	–	–	–	–	–	MS
73	1874	Fragranyl 3-methyl butyrate	0.4 ± 0	–	–	–	–	–	MS
74	1880	Benzyl 2-methylbutyrate	–	0.1	0.1	0.2	–	–	MS
75	1893	Geranyl isovalerate	1.23 ± 0.05	0.1	0.4	–	0.5	–	MS
77	1904	Geranyl 2-methyl butyrate	–	0.1	0.1	–	–	–	MS
78	1933	Neryl valerate	–	0.3	1.0	–	0.7	–	MS
79	2008	Caryophyllene oxide	0.33 ± 0	1.7	4.8	5.1	3.0	2.6	*t*_R_, MS
80	2037	Salvial-4(14)-en-1-one	–	0.2	–	–	–	0.3	MS
81	2050	(*E*)-Nerolidol	0.2 ± 0	–	–	–	–	–	*t*_R_, MS
82	2071	Humulene epoxide-II	–	0.2	–	–	–	0.2	MS
83	2080	Junenol (=Eudesm-4(15)-en-6-ol)	–	0.2	–	–	–	0.3	MS
84	2084	Octanoic acid	–	0.2	–	–	–	–	*t*_R_, MS
85	2096	Elemol	–	0.3	0.6	1.0	1.8	–	*t*_R_, MS
86	2096	*cis*-Sesquisabinene hydrate	0.4 ± 0.08	–	–	–	–	–	MS
87	2113	Cumin alcohol	–	0.1	–	–	–	–	*t*_R_, MS
88	2131	Hexahydrofarnesyl acetone	–	0.1	–	0.5	–	0.2	MS
89	2144	Spathulenol	0.2 ± 0.08	0.4	–	0.4	–	0.3	MS
90	2186	Eugenol	–	–	–	0.7	–	–	*t*_R_, MS
91	2192	Nonanoic acid	–	0.1	–	–	–	–	*t*_R_, MS
92	2239	Carvacrol	–	4.0	0.7	0.8	1.1	0.6	*t*_R_, MS
93	2257	β-Eudesmol	–	2.4	10.2	4.3	6.6	9.1	*t*_R_, MS
94	2264	Intermedeol	2.0 ± 0	–	–	–	–	–	MS
95	2298	Decanoic acid	–	–	–	–	–	0.2	*t*_R_, MS
96	2312	9-Geranyl-*p*-cymene	0.5 ± 0.08	–	–	–	–	–	MS
97	2324	Caryophylla-2(12),6(13)-dien-5α-ol (=*Caryophylladienol II*)	–	–	0.4	0.5	–	–	MS
98	2341	9-(15,16-dihydro-15-methylene)-Geranyl-*p*-cymene	0.7 ± 0	0.7	8.7	6.0	9.9	10.3	MS
99	2392	Caryophylla-2(12),6-dien-5β-ol (=*Caryophyllenol II*)	–	0.3	1.0	0.7	–	0.8	MS
100	2396	γ-Dodecalactone	–	0.1	–	–	–	–	MS
101	2430	Chamazulene	0.6 ± 0.08	0.3	3.9	2.8	3.1	2.9	*t*_R_, MS
102	2509	Methyl linoleate	–	–	0.4	–	–	–	*t*_R_, MS
103	2931	Hexadecanoic acid	–	–	1.3	0.4	–	1.3	*t*_R_, MS
		Total identified	93.15 ± 0.97	98.6	89.7	91.7	90.1	90.2	

a Relative retention indices calculated against *n*-alkanes on the HP Innowax column.

b Mean % calculated from flame ionization detector (FID) data ± SD (*n* = 3 for A_sd_ EO).

c Identification method; *t*_R_, identification based on the retention times of genuine compounds on the HP Innowax column; MS, identified based on computer matching of the mass spectra with those of the Wiley and MassFinder libraries and comparison with literature data.

#### 2.2.3 Identification of essential oil constituents

The identification of the EO components was carried out by co-injection with available standard compounds, which were purchased from commercial sources or obtained from natural sources. In addition, computer matching against commercial, Wiley GC/MS Library, MassFinder software 4.0 (McLafferty and Stauffer, 1989; Hochmuth, 2008), and in-house “Başer Library of Essential Oil Constituents” built up by genuine compounds and components of known oils, as well as MS literature data, was used for the identification. A C_9_–C_40_*n*-alkane standard solution (Fluka, Buchs, Switzerland) was used to spike the samples for the determination of chromatographic retention indices (RIs).

### 2.3 Laboratory bioassays

#### 2.3.1 Short-range attraction bioassays with *C. capitata*

Bioassays were conducted using sterile male *C. capitata*, consistent with the insect source, rearing procedures, and short-range attraction methods detailed by Tabanca et al. (2020). All experiments took place at room temperature within screened cages (20.3 cm^3^, BioQuip Products, Rancho Dominguez, CA, USA). Fifty flies, 7–15 days old, were introduced into each cage 1 hour before starting an experiment. Each assay began by placing two Petri dishes (53-mm diameter × 12-mm height) containing a Whatman #1 filter paper disk (3.5-cm diameter) soaked with 10 μL of a 10% dilution of the test compound in acetone on two opposing sides of the cages spaced 25 cm apart. All flies were discarded and not reused after assay termination. Bioassay development was divided into three parts.

Experiment 1: In the first set of preliminary bioassays, single, separate cages were used to evaluate the *C. capitata* response to six different combinations: 1) tea tree oil (TTO) versus acetone, 2) A_sd_ versus acetone, 3) A_1_ versus acetone, 4) A_2_ versus acetone, 5) A_3_ versus acetone, 6) A_4_ versus acetone, and 7) A_5_ versus acetone.
Experiment 2: In the second set of bioassays, cages were used to evaluate the *C. capitata* response to 1) TTO versus acetone, 2) α-thujone versus acetone, and 3) α,β-thujone versus acetone
Experiment 3: In the third set of bioassays, cages were used to evaluate the *C. capitata* response to 1) TTO versus α-thujone, 2) TTO versus α,β-thujone, and 3) α-thujone versus α,β-thujone.

Fly responses were measured by counting the number of flies within the Petri dish at 5, 10, 15, 30, 45, 60, 75, and 90 minutes. The first set of preliminary bioassays tested one cage of each combination. Each test in the second and third sets of bioassays was replicated 12 times, with cage positions randomized between runs. TTO [*M. alternifolia* (Maiden & Betche) Cheel.] was included as a positive control due to its powerful short-range attraction to male *C. capitata* (Shelly and Epsky, 2015; Tabanca et al., 2020).

#### 2.3.2 Toxicity of thujones against female adult *A. suspensa*

The colony of Caribbean fruit fly, *A. suspensa*, was maintained in rearing cages (30 × 30 × 30 cm) in an insectary in the toxicology laboratory in the Horticultural Research Station in the USDA-ARS at Miami, Florida, since August 2020. A*. suspensa* originated from field-collected guava fruits that were infested by *A. suspensa* in 2020, and since then, it has been maintained on an artificial diet in the insectary under 26.0°C ± 0.5°C, 70% ± 5% relative humidity, and 12:12 (light:dark), without exposure to any insecticides.

Topical bioassays using thoracic application to adult female *A. suspensa* were conducted to determine the toxicities of α-thujone and α,β-thujone under laboratory conditions similar to those mentioned above in the toxicology laboratory. Stock solutions of α-thujone and α,β-thujone were first prepared by dissolving 100 μg of each compound in 1 μL of acetone to establish a 100 μg/μL solution. Stock solution of each compound was then diluted serially to establish 0.05, 0.1, 0.2, 0.25, 0.5, and 0.75 μg/μL solutions, and each dilution was used in topical bioassays.

The procedure of the topical bioassay was similar to our previous study (Tok et al., 2023). To collect the synchronized female adult *A. suspensa* for the topical bioassay, pupae of *A. suspensa* were collected from rearing cages in the insectary and placed in a tray inside a screen cage (30 cm × 30 cm × 30 cm) under laboratory conditions. After adult emergence, female adults (<3 days old) were then collected using an aspirator into a plastic vial (3 cm in diameter × 8 cm in height). To begin the bioassay, vials containing female adults were placed in a refrigerator first at 10°C for 5 minutes to calm the flies, and calmed flies were then removed from the refrigerator to a Petri dish for the topical application. On each fly, a repeating dispenser equipped with a gastight and microliter syringe (50 μL) (PB600, Hamilton Company, Reno, NV, USA) was used to apply a 1-μL dilution at each concentration of each compound on the dorsal thorax of the calmed adult flies. After topical application, the adult flies were immediately transferred into a plastic cup (6 cm in diameter × 7.4 cm in height) and covered with a mesh screen for post-treatment observation. A block of sugar and yeast hydrolysate mixture (4:1 per weight) (1 cm^3^) and a block of water agar (1 cm^3^) were placed on top of the mesh screen to supply the food and water for the tested flies. To remove the chilling effect from the treatment, only adult flies recovered from chill were used for the experiment after a topical bioassay. After 24 hours, the numbers of live and dead flies were documented, and the mortality of *A. suspensa* in each treatment was calculated. Untreated female adults and those treated with acetone alone were used as controls. For each dilution of α-thujone and α,β-thujone, 10–15 female adult flies were treated, and for the control treatment, 10 female adult flies were treated with acetone. Each treatment was replicated three times. In total, 260 female flies were used in the evaluation of thujones.

### 2.4 Statistical analysis

Principal component analysis (PCA) and hierarchical cluster analysis (HCA) were carried out using the JMP Pro version 18.2.1 (SAS Institute, Cary, NC, USA). The covariance data matrix for the volatile compounds was an X × X matrix (28 individual compounds × 6 samples = 168 data). The PCA was performed using the JMP default standard method. The HCA was performed using Ward’s method. Short-range attraction data in the second set of bioassays were analyzed using JMP Pro 16 (SAS Institute). All short-range attraction data were found to meet assumptions of normality.

A mixed model was utilized for the separate analysis of the effect of factors on fly response to individual compounds within each of the six combinations tested. Fly age and time (minutes) were treated as fixed effects, while replicate was treated as a random effect. Time was treated as a repeated structure, with replicate as the subject. A compound symmetry structure was chosen, and Tukey’s pairwise comparisons of fly responses were performed between time points for each compound. Multiple comparison tests were used to test for differences in male responses at each time point to compounds within the six comparison tests.

The mortality data of *A. suspensa* in toxicity bioassays were used to calculate the lethal dose (LD_50_ and LD_99_) for α-thujone and α,β-thujone. Mortality data for each treatment were corrected by mortalities in the untreated control using Abbott’s formula (Abbott, 1925) before the analysis. A probit analysis was then used to calculate the lethal dose corresponding to a 50% and 99% reduction (LD_50_ and LD_99_) in *A. suspensa*’s survival based on the regression curve. The statistical analysis was performed using SAS (version 9.4, SAS Institute Inc., Cary, NC, USA).

## 3 Results

### 3.1 Chemical composition of *A. absinthium* essential oils

Essential oil yield is a key factor for economically important EOs such as *A. absinthium* EO. The conventional hydrodistillation method was used for yielding. Pale yellow EOs were obtained with yields of 1.0 to 3.5 mL/kg (0.1% to 0.35%) for A_1_ to A_5_ samples. According to the European Pharmacopoeia, the EO yield for *A. absinthium* should not be less than 2 mL/kg (0.2%), and the wormwood herb consists of basal leaves, slightly leafy, flowering tops, or a mixture of these dried organs (European Pharmacopeia, 2008). The highest EO yield was obtained when plant material was collected at full bloom and distilled shortly after collection [e.g., Asd sample, 3.8 mL/kg (0.38%), **Supplementary Table S1**]. EO production is highly influenced by resources, storage conditions (packed or unpacked), age of plants, and plant population density (flowers, leaves, stems, and branches).

**Figure 2:**
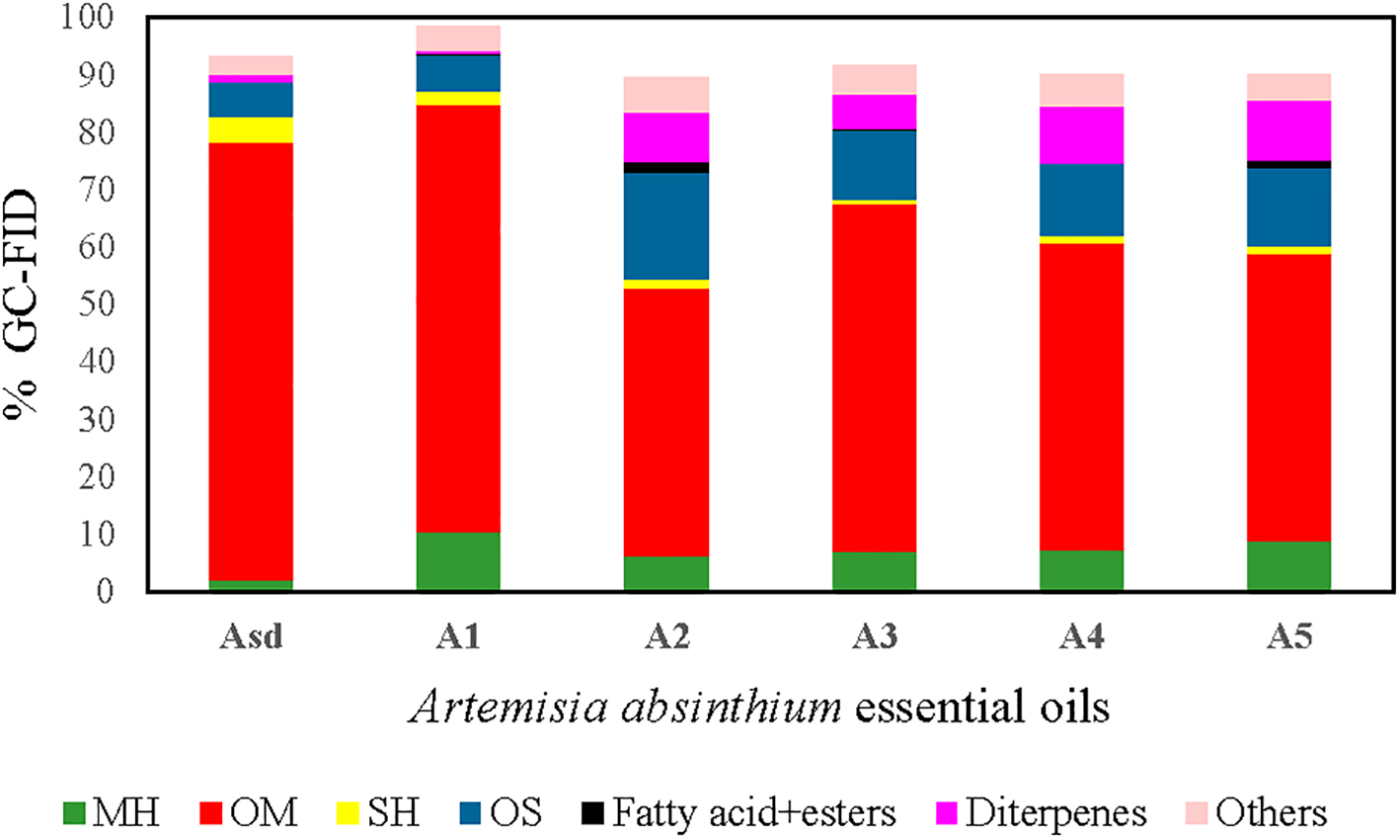
Percent composition of terpene groups in the *Artemisia absinthium* EOs: MH (monoterpene hydrocarbons), OM (oxygenated monoterpenes), SH (sesquiterpene hydrocarbons), OS (oxygenated sesquiterpenes), aliphatic fatty acid+esters, diterpenes, and others. EOs, essential oils.

GC–FID and GC–MS techniques were used to analyze the chemical profile of *A. absinthium* EOs. A total of 103 compounds constituting 89.7% to 98.6% of the total oil were detected in the six *A. absinthium* samples. The identified compounds, their relative percentages, and retention indices are listed in **Table 1** in order of their elution time on the polar column. The EOs are composed of monoterpene hydrocarbons (2.03%–10.5%), oxygenated monoterpenes (46.5%–76.22%), sesquiterpene hydrocarbons (0.9%–4.64%), oxygenated sesquiterpenes (6.03%–18.5%), oxygenated diterpenes (0.7%–10.3%), fatty acids+esters (0%–1.7%), and other non-terpenoids (3.03%–6.2%) (**Figure 2**). In examined oils, β-thujone (39.87%), (*Z*)-β-ocimene epoxide (17.87%), and sabinyl acetate (13.87%) were the major constituents of A_sd_ oil, followed by intermedeol (2.0%), β-selinene (1.8%), neryl isovalerate (1.67%), linalool (1.67%), geranyl isovalerate (1.23%), α-thujene (1.17%), and neryl propionate (1.1%); in contrast, A_1_–A_5_ EOs were dominated by camphor (30.5%–50.8%), followed by β-thujone (0%–28.4%), β-eudesmol (2.4%–10.2%), and diterpene 9-(15,16-dihydro-15-methylene)-geranyl-*p*-cymene (0.7%–10.3%). The remaining most common compounds that occurred were caryophyllene oxide (1.7%–5.1%), carvacrol (0.7%–4%), chamazulene (0.3%–3.9%), *p*-cymene (2.1%–3.9%), camphene (1.0%–3.2%), terpinen-4-ol (1.2%–2.8%), and 2-methylbutyl isovalerate (0.7%–2.2%). From all the identified compounds, β-thujone was determined as a major compound in A_sd_ EO, whereas camphor was the main compound in five samples, A_1_ to A_5_ EOs. β-Thujone and α-thujone were assessed only as A_sd_ and A_1_ EOs, and camphor was absent from the A_sd_ EO. In addition, (*Z*)-β-ocimene epoxide, sabinyl acetate, β-eudesmol, and pleasant floral compounds, neryl propionate, neryl isovalerate, nerol, and (*E*)-nerolidol, were detected only in A_sd_ EO. The situation was similar with A_1_ to A_5_ samples: compounds camphene, 2-methylbutyl isovalerate, α-terpineol, borneol, and carvacrol were present in 1% and over only in A_1_ to A_5_ samples, while γ-terpinene, *p*-cymene, terpinen-4-ol, caryophyllene oxide, 9-(15,16-dihydro-15-methylene)-geranyl-*p*-cymene, and chamazulene were found in all six samples (A_sd_ and A_1_ to A_5_).

### 3.2 Multivariate analysis of the *A. absinthium* essential oils

Multivariate statistical analysis was subjected to PCA and HCA to visualize the similarities and differences among the *A. absinthium* samples based on volatile compounds by reducing the number of dimensions without much loss of information. PCA and HCA were performed on the values of the relative volatile concentrations ≥1.0% using the correlation matrix with no rotation and applied to clarify the relationship among the *Artemisia* samples. PCA resulted in the first two components (PC1 and PC2) with eigenvalues greater than one, with PC1 55.1% and PC2 27.5% of variance, respectively. Bartlett’s sphericity and the Kaiser–Meyer–Olkin (KMO) test were performed to validate the dataset’s suitability and sampling adequacy for a suitable PCA application. The results of Bartlett’s sphericity test (*p* < 0.05) and KMO test (KMO > 0.5) indicated that the dataset was suitable and adequate, respectively (**Table 2**).

**Table 2:** Eigenvalues, variability, cumulative variability, Bartlett’s sphericity test, and Kaiser–Meyer–Olkin (KMO) test are associated with each principal component (PC).

Variables	PC1	PC2	PC3	PC4	PC5
Eigenvalues	15.42	7.70	2.54	1.45	0.89
Variability (%)	55.06	27.51	9.09	5.17	3.17
Cumulative variability (%)	55.06	82.57	91.66	96.83	100
Bartlett’s sphericity test	*p* < 0.0001, there is at least one of the correlations between the variables that is significantly different from 0				
Kaiser–Meyer–Olkin (KMO) test	0.579, KMO > 0.5 is deemed adequate for performing PCA				

**Figure 3:**
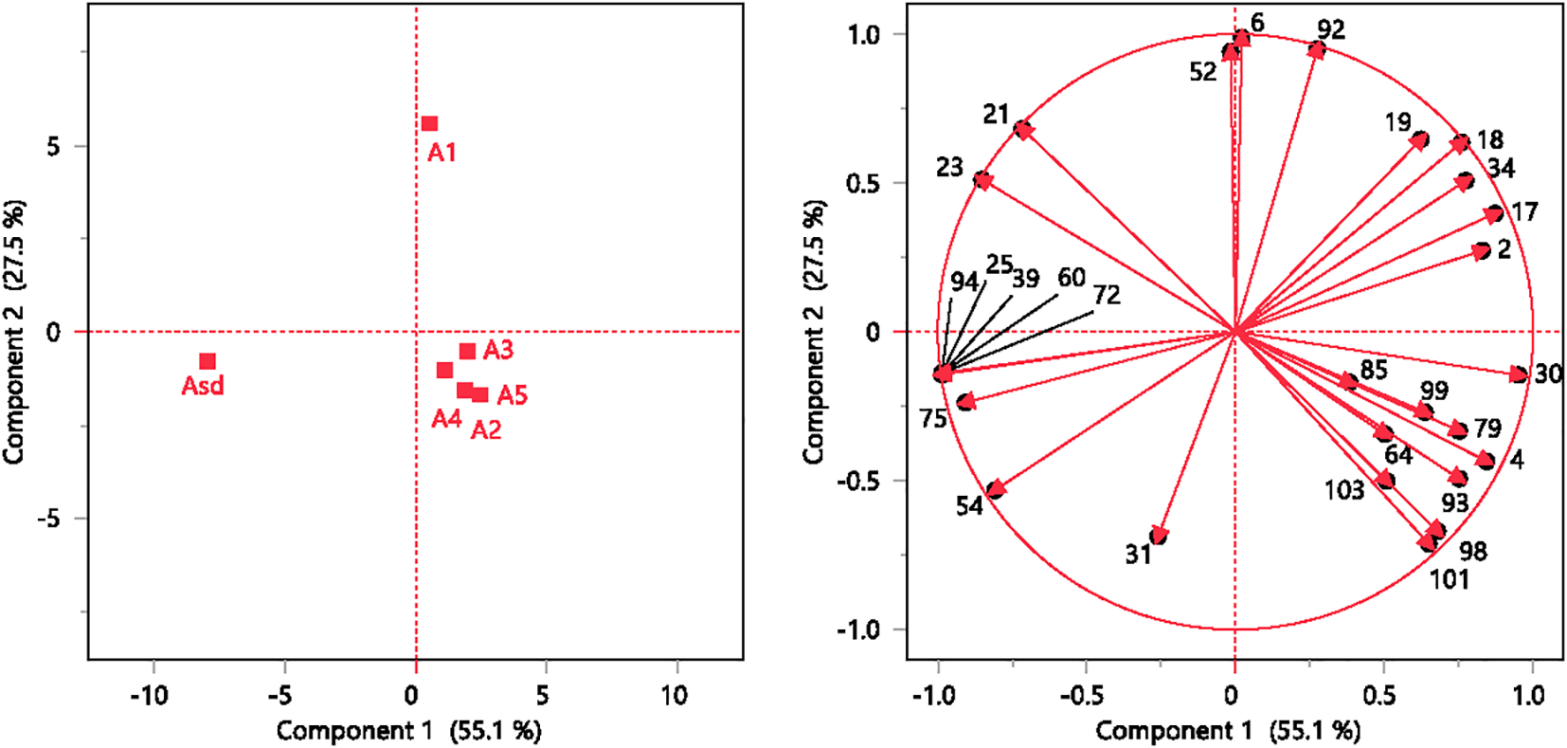
Results of the PCA. PC1 vs. PC2 score plots for the investigated observations (left). PC1 vs. PC2 loading plots for the investigated variables (right). Compound numbers are listed in **Table 1**. PCA, principal component analysis.

PCA separated the *A. absinthium* samples into three groups based on their volatile composition. Authenticated sample A_sd_ is located in the lower quadrant at the left-hand side of the plot, displaying negative values for both PC1 and PC2, in contrast to the A_1_ sample, which is present in the right-hand side of the plot, showing positive values for both PC1 and PC2. Commercial samples A_1_ to A_5_ are clustered together on the positive side of PC1 and the negative side of PC2 (**Figure 3**). Factor loading and high cos^2^ values determine a strong positive or negative correlation between observations and variables, which is therefore considered an important observation or variable for judgment. Among the 28 Volatile Organic Compounds (VOCs), the highest cos^2^ values were in PC1: sabinyl acetate (0.972), (*Z*)-β-ocimene epoxide (0.970), camphor (0.907), neryl propionate (0.970), neryl isovalerate (0.970), intermedeol (0.970), geranyl isovalerate (0.822), γ-terpinene (0.761), β-thujone (0.724), and camphene (0.712). For PC2, the VOCs were β-pinene (0.977), carvacrol (0.903), and germacrene D (0.885) (**Table 3**).

**Table 3:** Factor loadings, contributions (%), and squared cosines (cos^2^) values.

#	Compounds	Factor loadings and contributions (%)		Squared cosines (cos^2^)	
		PC1	PC2	PC1	PC2
2	α-Pinene	**0.829**	0.273	**0.687**	0.075
4	Camphene	**0.844**	−0.435	**0.712**	0.189
6	β-Pinene	0.022	**0.989**	0.000	**0.977**
17	γ-Terpinene	**0.872**	0.397	**0.761**	0.158
18	*p*-Cymene	**0.760**	**0.636**	**0.577**	0.404
19	2-Methylbutyl isovalerate	**0.623**	**0.646**	0.389	0.418
21	α-Thujone	**−0.716**	**0.682**	**0.512**	0.465
23	β-Thujone	**−0.851**	**0.511**	**0.724**	0.261
25	(*Z*)-β-Ocimene epoxide	**−0.985**	−0.139	**0.970**	0.019
30	Camphor	**0.952**	−0.145	**0.907**	0.021
31	Linalool	−0.260	**−0.687**	0.067	0.472
34	Terpinen-4-ol	**0.773**	**0.507**	**0.598**	0.257
39	Sabinyl acetate	**−0.986**	−0.143	**0.972**	0.021
52	Germacrene D	−0.016	**0.941**	0.000	**0.885**
54	β-Selinene	**−0.808**	**−0.532**	**0.653**	0.283
60	Neryl propionate	**−0.985**	−0.139	**0.970**	0.019
64	Perilla aldehyde	**0.502**	−0.344	0.252	0.118
72	Neryl isovalerate	**−0.985**	−0.139	**0.970**	0.019
75	Geranyl isovalerate	**−0.907**	−0.238	**0.822**	0.056
79	Caryophyllene oxide	**0.752**	−0.332	**0.565**	0.111
85	Elemol	0.383	−0.170	0.147	0.029
92	Carvacrol	0.276	**0.950**	0.076	**0.903**
93	β-Eudesmol	**0.752**	−0.493	**0.566**	0.243
94	Intermedeol	**−0.985**	−0.139	**0.970**	0.019
98	9-(15,16-dihydro-15-methylene)-Geranyl-*p*-cymene	**0.680**	**−0.670**	0.462	0.449
99	Caryophylla-2(12),6-dien-5β-ol (=*Caryophyllenol II*)	**0.637**	−0.270	0.406	0.073
101	Chamazulene	**0.650**	−0.712	0.422	**0.507**
103	Hexadecanoic acid	**0.508**	−0.501	0.258	0.251

The contributions ≥0.5 are highlighted in bold. The highest cos^2^ values ≥0.5 are highlighted in bold.

**Figure 4:**
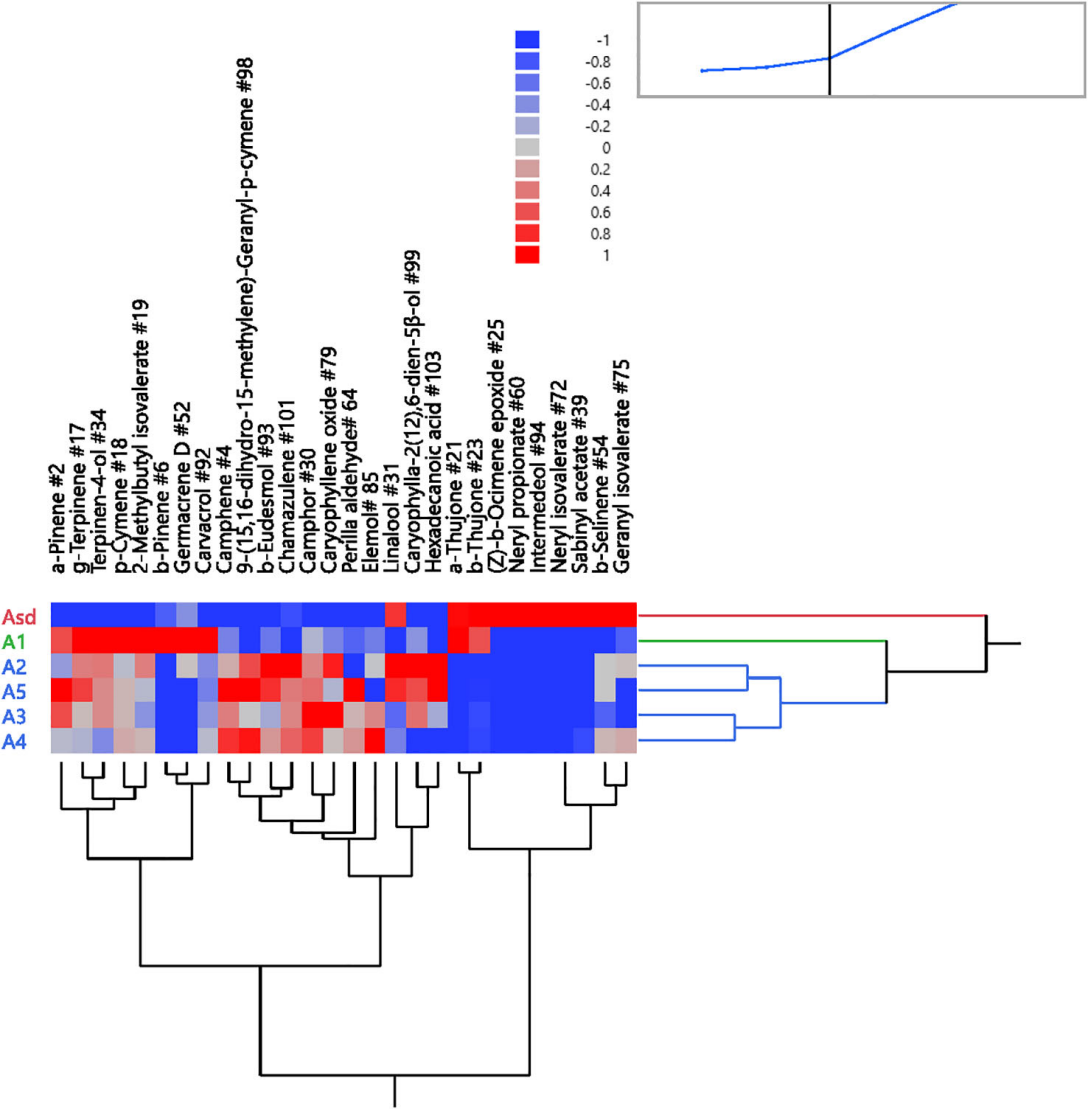
Two-way dendrogram of the hierarchical cluster analysis (HCA) was performed on the chemical compositions of six *Artemisia absinthium* samples (A_sd_ and A_1_ to A_5_). The color box indicates the abundance of each compound from **Table 1**. Red represents a high density of compounds, and blue represents low density.

The dendrogram graph generated by HCA based on the differences in EO components of the six *A. absinthium* samples agreed with the PCA scatter plots of the same samples. *A. absinthium* samples fell into three clusters. Cluster 1 encompassed A_sd_ with relatively high values of components β-thujone (39.87%), (*Z*)-β-ocimene epoxide (17.87%), and sabinyl acetate (13.87%). Cluster 2, comprising A_1_, was characterized by high camphor (30.5%) and β-thujone (28.4%), and samples A_2_ to A_5_ were embedded in cluster 3 (**Figure 4**) with high camphor (40.1%–50.8%), 9-(15,16-dihydro-15-methylene)-geranyl-*p*-cymene (6.0% to 10.3%), and β-eudesmol (4.3% to 10.2%). PCA and HCA revealed that *A. absinthium* samples from various sources were effectively identified and classified.

### 3.3 Behavioral responses of the sterile male medflies to *A. absinthium* oils in short-range assays

#### 3.3.1 Preliminary short-range assays: *A. absinthium* attraction

In our initial short-range assays, male *C. capitata* showed varied attraction to *A. absinthium* EOs. TTO, used as a positive control, was the most attractive after 30 minutes, followed by samples A_1_, A_sd_, A_5_, A_2_, A_4_, and A_4_ in decreasing order of attractiveness (**Supplementary Figure S1**). The attraction pattern for A_sd_ changed over time and captured more flies after 30 minutes. Based on the two-way hierarchical clustering dendrogram and heat map correlation, it indicates that α,β-thujones are higher than the mean levels (**Figure 4**). Therefore, we presumed that α,β-thujones may be the main components contributing to the bioactivity mediated by male *C. capitata*, and further bioassays were carried out to compare thujones. Additionally, unidentified components may also have contributed to this attraction but were not tested in this study.

#### 3.3.2 Bioassays with tea tree oil, α-thujone, and α,β-thujone

We then conducted a second set of bioassays to compare *C. capitata* response to TTO, α-thujone, and α,β-thujone against an acetone control (**Supplementary Table S2**).

##### 3.3.2.1 Tea tree oil and acetone

**Figure 5:**
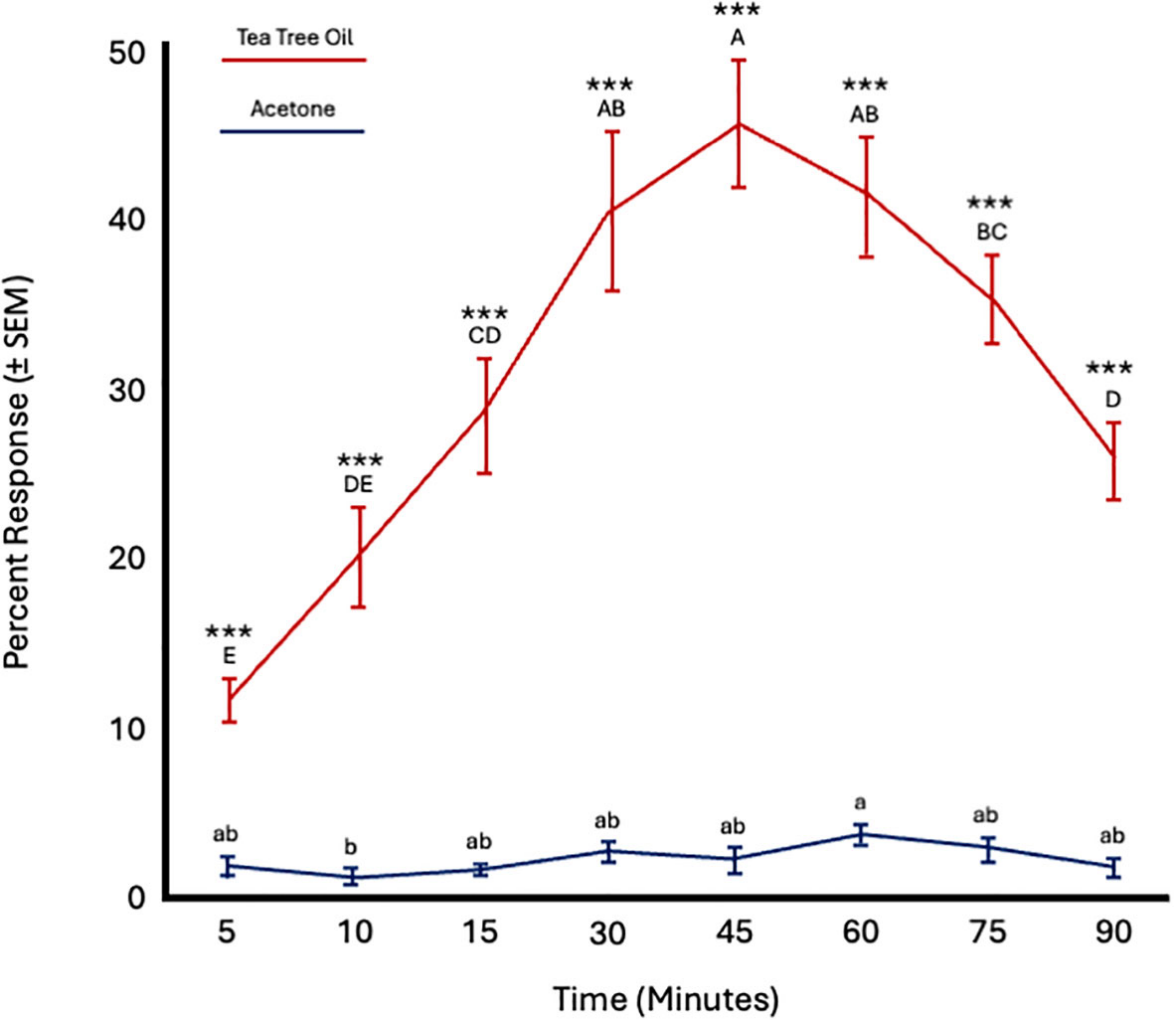
Percent attraction of sterile male *Ceratitis capitata* to tea tree oil vs. acetone control in small cage bioassays. Fifty flies were tested per cage across 12 replicates (*n* = 12). An asterisk (*p ≤ 0.05; **p ≤ 0.01) indicates a significant difference between responding flies at a specific time point (matched pairs t-test, *p* < 0.05). Means sharing the same letter are not significantly different within each test compound (mixed model: fly age and time as fixed effects, replicate as random effect, and time as repeated structure with replicate as subject; compound symmetry structure; Tukey's pairwise comparisons, *p* < 0.05).

Fly age did not significantly affect responses to TTO (*F* = 2.59; *df* = 5; *p* = 0.14) or acetone (*F* = 0.64; *df* = 5; *p* = 0.67). However, time significantly influenced responses to both tea tree oil (*F* = 32.15; *df* = 7; *p* < 0.0001) and acetone (*F* = 2.75; *df* = 7; *p* = 0.01). Attraction to tea tree oil increased from 5 to 45 minutes and then decreased from 60 to 90 minutes. More flies were attracted to tea tree oil at all time points (**Figure 5**; **Table 4**).

**Table 4:** Statistical comparisons between treatments at each time point in short-range bioassays.

Time	TTO vs. acetone	α-Thujone vs. acetone	αβ-Thujone vs. acetone	TTO vs. α-thujone	TTO vs. αβ-thujone	αβ-Thujone vs. α-thujone
5	*t* = −7.96 *df* = 11 *p* < 0.0001	*t* = −2.76 *df* = 11 *p* = 0.018	*t* = 2.46 *df* = 11 *p* = 0.032	*t* = −0.13 *df* = 11 *p* = 0.9	*t* = −0.32 *df* = 11 *p* = 0.76	*t* = 0.97 *df* = 11 *p* = 0.35
10	*t* = 6.22 *df* = 11 *p* < 0.0001	*t* = 2.21 *df* = 11 *p* = 0.049	*t* = 3.80 *df* = 11 *p* = 0.029	*t* = 0.07 *df* = 11 *p* = 0.95	*t* = −0.96 *df* = 11 *p* = 0.36	*t* = 0.94 *df* = 11 *p* = 0.36
15	*t* = 7.74 *df* = 11 *p* < 0.0001	*t* = −3.15 *df* = 11 *p* = 0.009	*t* = 3.81 *df* = 11 *p* = 0.029	*t* = −0.39 *df* = 11 *p* = 0.71	*t* = −1.02 *df* = 11 *p* = 0.33	*t* = 1.04 *df* = 11 *p* = 0.32
30	*t* = 7.62 *df* = 11 *p* < 0.0001	*t* = 3.33 *df* = 11 *p* = 0.007	*t* = −4.13 *df* = 11 *p* = 0.002	*t* = 2.46 *df* = 11 *p* = 0.03	*t* = −1.94 *df* = 11 *p* = 0.08	*t* = 1.4 *df* = 11 *p* = 0.19
45	*t* = 11.75 *df* = 11 *p* < 0.0001	*t* = 3.61 *df* = 11 *p* = 0.004	*t* = 4.33 *df* = 11 *p* = 0.001	*t* = 3.91 *df* = 11 *p* = 0.002	*t* = −2.76 *df* = 11 *p* = 0.02	*t* = 1.27 *df* = 11 *p* = 0.89
60	*t* = 9.62 *df* = 11 *p* < 0.0001	*t* = −3.36 *df* = 11 *p* = 0.006	*t* = 3.94 *df* = 11 *p* = 0.002	*t* = 4.08 *df* = 11 *p* = 0.002	*t* = −3.56 *df* = 11 *p* = 0.004	*t* = 0.72 *df* = 11 *p* = 0.49
75	*t* = −11.49 *df* = 11 *p* < 0.0001	*t* = 4.09 *df* = 11 *p* = 0.002	*t* = −4.53 *df* = 11 *p* < 0.0001	*t* = 6.82 *df* = 11 *p* < 0.0001	*t* = −4.98 *df* = 11 *p* = 0.0004	*t* = 1.15 *df* = 11 *p* = 0.28
90	*t* = 10.38 *df* = 11 *p* < 0.0001	*t* = 3.28 *df* = 11 *p* = 0.018	*t* = 6.04 *df* = 11 *p* < 0.0001	*t* = 5.79 *df* = 11 *p* < 0.0001	*t* = −4.74 *df* = 11 *p* = 0.0006	*t* = 0.62 *df* = 11 *p* = 0.55

Multiple comparison tests, *t*-test.

##### 3.3.2.2 α-Thujone and acetone

**Figure 6:**
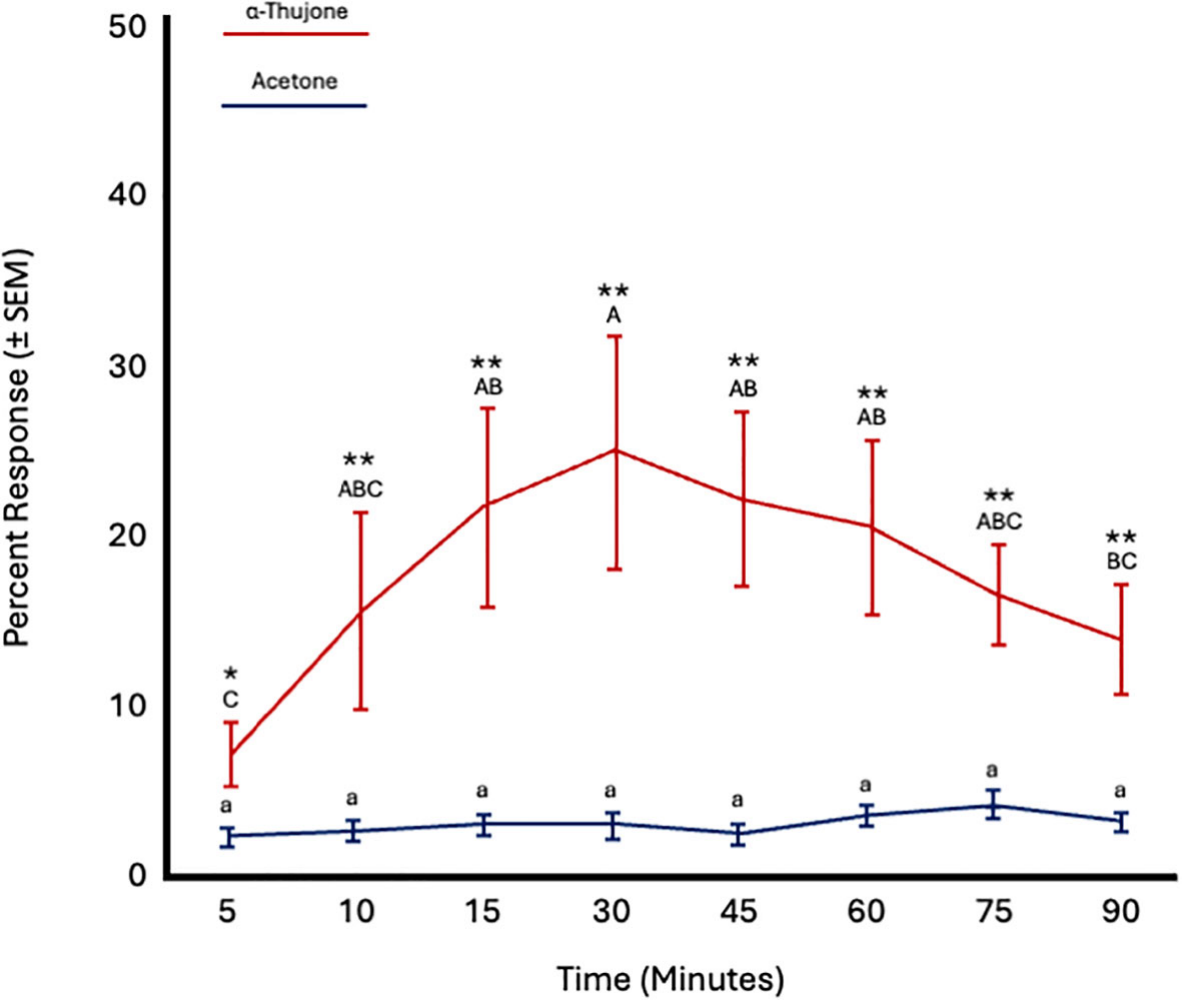
Percent attraction of sterile male *Ceratitis capitata* to α-thujone vs. acetone control in small cage bioassays. Fifty flies were tested per cage across 12 replicates (*n* = 12). An asterisk (*p ≤ 0.05; **p ≤ 0.01) indicates a significant difference between responding flies at a specific time point (matched pairs t-test, *p* < 0.05). Means sharing the same letter are not significantly different within each test compound (mixed model: fly age and time as fixed effects, replicate as random effect, and time as repeated structure with replicate as subject; compound symmetry structure; Tukey's pairwise comparisons, *p* < 0.05).

Similarly, fly age did not significantly impact responses to α-thujone (*F* = 1.09; *df* = 5; *p* = 0.45) or acetone (*F* = 0.89; *df* = 5; *p* = 0.54). There was a significant effect of time on the response to α-thujone (*F* = 6.53; *df* = 7; *p* < 0.0001), but not to acetone (*F* = 1.07; *df* = 7; *p* = 0.39). Attraction to α-thujone increased from 5 to 30 minutes and then decreased from 45 to 90 minutes. More flies were attracted to α-thujone at all time points (**Figure 6**; **Table 4**).

##### 3.3.2.3 α,β-Thujone and acetone

**Figure 7:**
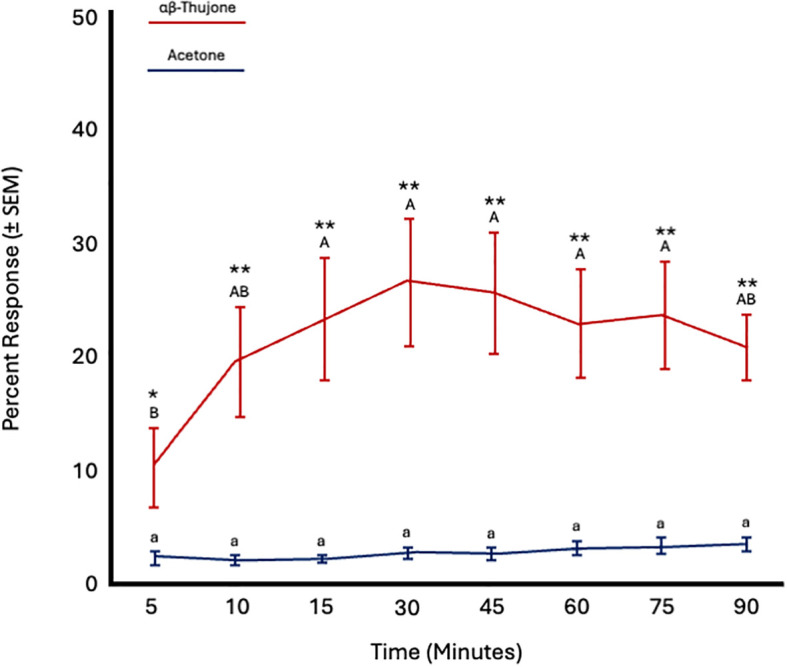
Percent attraction of sterile male *Ceratitis capitata* to αβ-thujone vs. acetone control in small cage bioassays. Fifty flies were tested per cage across 12 replicates (*n* = 12). An asterisk (*p ≤ 0.05; **p ≤ 0.01) indicates a significant difference between responding flies at a specific time point (matched pairs t-test, *p* < 0.05). Means sharing the same letter are not significantly different within each test compound (mixed model: fly age and time as fixed effects, replicate as random effect, and time as repeated structure with replicate as subject; compound symmetry structure; Tukey's pairwise comparisons, *p* < 0.05).

Fly age had no significant effect on responses to α,β-thujone (*F* = 0.55; *df* = 5; *p* = 0.73) or acetone (*F* = 0.43; *df* = 5; *p* = 0.81). However, time significantly affected the response to α,β-thujone (*F* = 4.04; *df* = 7; *p* = 0.0008), but not to acetone (*F* = 1.06; *df* = 7; *p* = 0.39). Attraction to α,β-thujone increased from 5 to 10 minutes and then remained constant from 10 to 90 minutes. More flies were attracted to α,β-thujone at all time points (**Figure 7**; **Table 4**).

##### 3.3.2.4 Bioassays with combinations of tea tree oil, α-thujone, and α,β-thujone

Our third set of bioassays examined *C. capitata* responses to three different combinations of these compounds.

##### 3.3.2.5 Tea tree oil versus α-thujone

**Figure 8:**
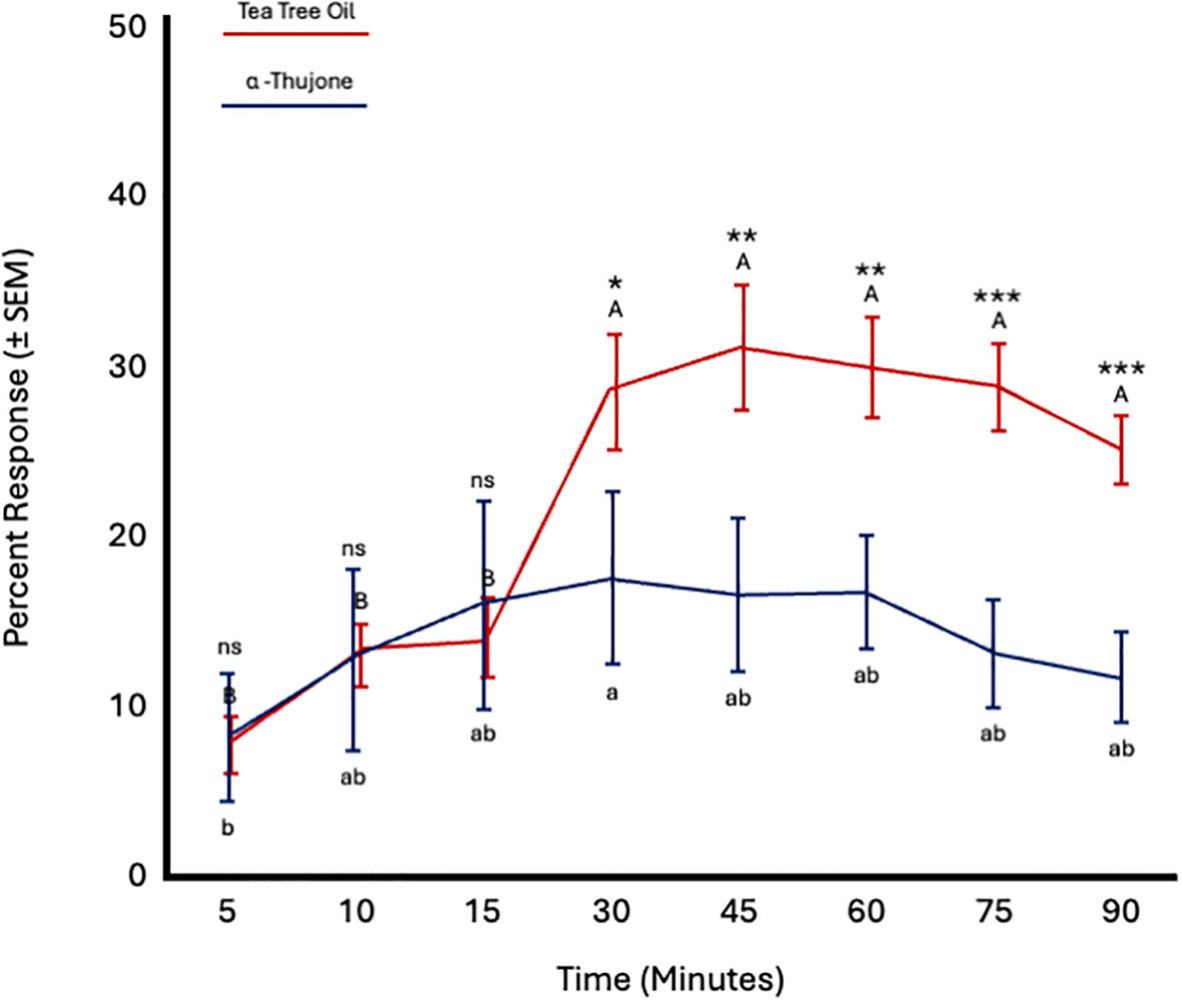
Percent attraction of sterile male *Ceratitis capitata* to tea tree oil vs. α-thujone in small cage bioassays. Fifty flies were tested per cage across 12 replicates (*n* = 12). An asterisk (*p ≤ 0.05; **p ≤ 0.01; ***p ≤ 0.001) indicates a significant difference between responding flies at a specific time point (matched pairs t-test, *p* < 0.05). Means sharing the same letter are not significantly different within each test compound (mixed model: fly age and time as fixed effects, replicate as random effect, and time as repeated structure with replicate as subject; compound symmetry structure; Tukey's pairwise comparisons, *p* < 0.05). ns: not significant, p-value≥0.05.

Fly age did not significantly affect responses to tea tree oil (*F* = 0.5; *df* = 5; *p* = 0.77) or α-thujone (*F* = 0.43; *df* = 5; *p* = 0.82). However, time significantly influenced responses to both TTO (*F* = 27.27; *df* = 7; *p* < 0.0001) and α-thujone (*F* = 2.62; *df* = 7; *p* = 0.018). Fly attraction to TTO increased from 15 to 30 minutes and remained constant thereafter until 90 minutes. Attraction to α-thujone increased from 5 to 10 minutes and then remained constant until 90 minutes. More flies were attracted to TTO from 30 to 90 minutes (**Figure 8**; **Table 4**).

##### 3.3.2.6 Tea tree oil versus α,β-thujone

**Figure 9:**
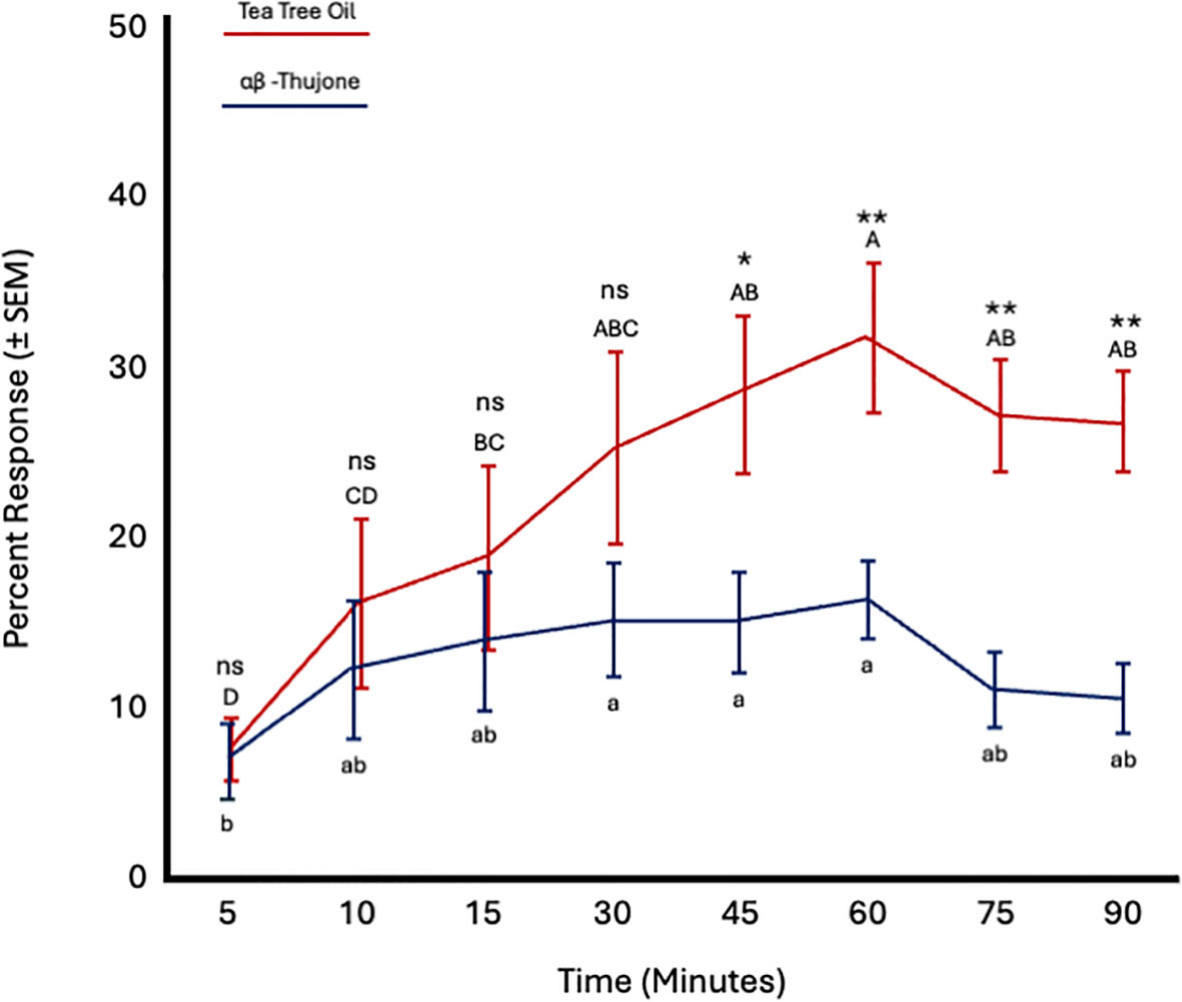
Percent attraction of sterile male *Ceratitis capitata* to tea tree oil vs. αβ-thujone in small cage bioassays. Fifty flies were tested per cage across 12 replicates (*n* = 12). An asterisk (*p ≤ 0.05; **p ≤ 0.01) indicates a significant difference between responding flies at a specific time point (matched pairs t-test, *p* < 0.05). Means sharing the same letter are not significantly different within each test compound (mixed model: fly age and time as fixed effects, replicate as random effect, and time as repeated structure with replicate as subject; compound symmetry structure; Tukey's pairwise comparisons, *p* < 0.05). ns: not significant, p-value≥0.05.

Fly age had no significant effect on responses to tea tree oil (*F* = 0.61; *df* = 5; *p* = 0.69) or α,β-thujone (*F* = 0.98; *df* = 5; *p* = 0.49). Conversely, time significantly affected responses to both TTO (*F* = 11.07; *df* = 7; *p* < 0.0001) and α,β-thujone (*F* = 3.56; *df* = 7; *p* = 0.002). Fly attraction to TTO increased from 15 to 60 minutes and remained constant from 60 to 90 minutes. Attraction to α,β-thujone increased from 5 to 10 minutes and remained constant from 15 to 90 minutes. More flies were attracted to tea tree oil than α,β-thujone from 45 to 90 minutes (**Figure 9**; **Table 4**).

##### 3.3.2.7 α-Thujone versus α,β-thujone

**Figure 10:**
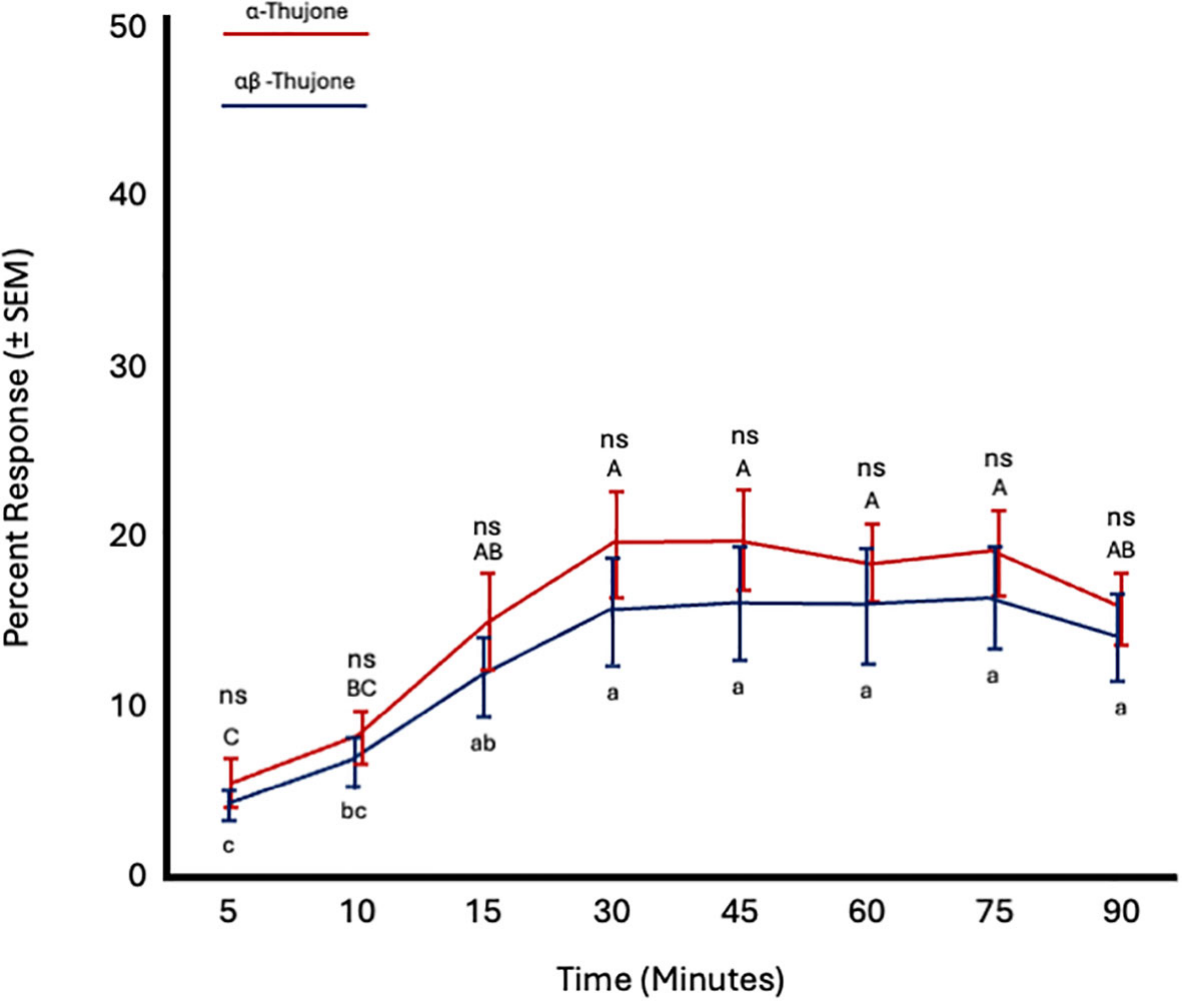
Percent attraction of sterile male *Ceratitis capitata* to α-thujone vs. αβ-thujone in small cage bioassays. Fifty flies were tested per cage across 12 replicates (*n* = 12). Means sharing the same letter are not significantly different within each test compound (mixed model: fly age and time as fixed effects, replicate as random effect, and time as repeated structure with replicate as subject; compound symmetry structure; Tukey's pairwise comparisons, *p* < 0.05). ns: not significant, p-value≥0.05.

In the final set of short-range bioassays, fly age did not significantly affect *C. capitata* response to either α-thujone (*F* = 0.77; *df* = 5; *p* = 0.6) or α,β-thujone (*F* = 0.75; *df* = 5; *p* = 0.61). However, time significantly influenced responses to both compounds (α-thujone: *F* = 9.7; *df* = 7; *p* < 0.0001; α,β-thujone: *F* = 9.46; *df* = 7; *p* < 0.0001). Attraction to both α-thujone and α,β-thujone increased from 5 to 15 minutes and then remained constant from 30 to 90 minutes. Flies were similarly attracted to both α-thujone and α,β-thujone at all time points (**Figure 10**; **Table 4**).

### 3.4 Exploring the effects of α,β-thujones on female caribflies

The present study showed that both α-thujone and αβ-thujone demonstrated strong toxicity to adult female *A. suspensa*. Our results showed that the median lethal doses (LD_50_) of α-thujone and α,β-thujone were 0.21 and 0.14 μg/μL, respectively, and the lethal doses responsible for a 99% reduction were 0.66 and 0.71 μg/μL, respectively (**Table 5**). Our results also showed that both untreated and acetone controls had 0% mortality in female adults. A dose of 0.14 μg of α,β-thujone was required to achieve 50% mortality of female adults, which was lower than the dose required by α-thujone, 0.21 μg/μL. However, a higher dose of α,β-thujone was required than that of α-thujone (**Table 5**).

**Table 5:** Lethal dose (LD_50_ and LD_99_) of α,β-thujone and α-thujone for the control of the adult female Caribbean fruit fly, *Anastrepha suspensa*, under laboratory conditions.

Compounds	n*	Slope ± SE	LD_50_ μg/μL	LD_99_ μg/μL	*χ* ^2^	*χ* ^2^	*p*
α-Thujone	160	4.64 ± 0.64	0.2068 (0.1815–0.2470)	0.6553 (0.4569-0.8042)	6.8827	4	0.1422
α,β-Thujone	100	3.28 ± 0.71	0.1394 (0.0984–0.1791)	0.7143 (0.3723-1.4756)	3.9562	4	0.4120

*Total number of female adult *A. suspensa* tested.

## 4 Discussion

### 4.1 Chemical survey

Literature surveys showed that the chemical composition and essential oil profiles of *A. absinthium* (wormwood) varied significantly among different populations and even within the same species (**Table 6**). This high level of “intraspecific variability” is a key indicator of a genetic component and ecological factors. Studies comparing *A. absinthium* from different European countries (e.g., Austria, France, Germany, Hungary, Italy, and Spain) found that their *A. absinthium* EOs were significantly different. Ecological factors such as altitude, temperature, and precipitation can influence the plant’s metabolism, leading to variations in the production of secondary metabolites. Studies comparing *A. absinthium* from different European countries (e.g., Austria, Belgium, Croatia, England, Estonia, Germany, Georgia, Hungary, Italy, Latvia, Lithuania, Moldova, Poland, Scotland, Serbia, Spain, and The Netherlands) found that their *A. absinthium* EOs showed significant qualitative and quantitative differences. Studies have identified several chemotypes based on the dominant compounds in *A. absinthium* EOs within any plant tissues, including i) thujone-rich chemotypes (Tucker et al., 1993; Nin and Bosetto, 1995; Juteau et al., 2003; Orav et al., 2006; Msaada et al., 2015; Llorens-Molina et al., 2016; Nguyen et al., 2018a, 2018; Radulovic et al., 2022; Raal et al., 2024; Tojic et al., 2025), ii) epoxyocimene+chrysanthenyl acetate or epoxyocimene+chrysanthenol (Carnat et al., 1992; Mucciarelli et al., 1995; Arino et al., 1999; Juteau et al., 2003; Llorens-Molina and Vacas, 2015; Julio et al., 2015; Llorens-Molina et al., 2016; Nguyen et al., 2018a, 2018; Politeo et al., 2023), iii) sabinyl acetate-rich chemotypes (Orav et al., 2006; Judzentiene and Budiene, 2010; Nguyen et al., 2018a; Kosakowska et al., 2025), and iv) mixed terpenoids (Rezaeinodehi and Khangoli, 2008; Taherkhani et al., 2013; Mihajilov-Krstev et al., 2014; Dhen et al., 2014; Riahi et al., 2015; Malaspina et al, 2025). Chrysanthenol chemotype was quite rare, and it was determined in wormwood oils from European countries, such as Hungary, Spain, and Poland (Bailen et al., 2013; Julio et al., 2015; Nguyen et al., 2018a; Kosakowska et al., 2025). Studies have also shown that *A. absinthium* chemical components are differentiated from different continents. For example, a study on *A. absinthium* growing in Italy found high concentrations of cis-epoxyocimene (24.8%) and trans-chrysanthenyl acetate (21.6%) (Mucciarelli et al., 1995), while a Cuban sample had bornyl acetate (23.02%) and terpinen-4-ol (8.15%) (Pino et al., 1997). The chamazulene-rich dark blue color of *A. absinthium* EOs was found in high concentrations in some of the samples, such as the eastern part of Turkey (17.8% and 28.6%) (Kordali et al., 2005; Bagci et al., 2010), Tunisia (5.98%-42.965) (Riahi et al., 2015), Algeria (10.05%) (Benmimoune et al., 2023), and Saudi Arabia (10.8%) (Aati et al., 2024). Another interesting compound, davanone, a sesquiterpene ketone, was found in 60.2% in Saudi Arabian *A. absinthium* EO (Aati et al., 2024), 35.88% in a Tunisian sample (Malaspina et al., 2025), and 16.4% in an Ethiopian sample (Tariku et al., 2011). This could be related to heat and drought stress. This explains why certain chemotypes are more prevalent in specific regions.

**Table 6:** Main components of essential oils from *Artemisia absinthium* samples.

Main components	Origin	Plant part used	Reference
*cis*-Chrysanthenol (69.01%), myrcene (10.40%), β-thujone (4.22%)	France	Aerial parts	Carnat et al., 1992
Sabinene (29.85%), myrcene (29.83%), α-phellandrene (19.02%)	United States	Leaves	Tucker et al., 1993
β-Thujone (33.11%), *cis*-chrysanthenyl acetate (32.75%)	United States	Commercial EO	Tucker et al., 1993
*cis*-Epoxyocimene (24.8%), *trans*-chrysanthenyl acetate (21.6%), camphor (17.1%), spathulenol (7.9%), caryophyllene oxide (10.0%)	Italy	Aerial parts	Mucciarelli et al., 1995
β-Thujone (0-49.21%), terpinen-4-ol (0%–28.82%)	Italy	Leaves+flowers	Nin and Bosetto, 1995
β-Thujone (0%–34.92%)	Austria	Leaves+flowers	Nin and Bosetto, 1995
β-Thujone (0.29%–49.80%)	Germany	Leaves+flowers	Nin and Bosetto, 1995
β-Thujone (5.91%–30.09%)	France	Leaves+flowers	Nin and Bosetto, 1995
β-Thujone (0%–69.68%), terpinen-4-ol (0.77%–11.64%)	United States	Leaves+flowers	Nin and Bosetto, 1995
Bornyl acetate (23.02%), terpinen-4-ol (8.15%)	Cuba	Stems+leaves	Pino et al., 1997
*cis*-Epoxyocimene (44.7%), *cis*-chrysanthenyl acetate (32.6%)	Spain	Leaves	Arino et al., 1999
*cis*-Epoxyocimene (37.3%), *cis*-chrysanthenyl acetate (43.0%)	Spain	Flowering tops	Arino et al., 1999
*cis*-Epoxyocimene (22.6%–49.7%), chrysanthenyl acetate (11.4%–36.7%)	France	Aerial parts	Juteau et al., 2003
β-Thujone (14.0%–48.6%), *cis*-epoxyocimene (6.1%–38.2%)	Croatia	Aerial parts	Juteau et al., 2003
Chamazulene (17.8%), nucifrol butanoate (8.2%)	Turkiye	Aerial parts	Kordali et al., 2005
β-Thujone (35.1%), *p*-cymene (16.5%), β-pinene (7.3%)	Iran	Aerial parts	Morteza-Semnani and Akbarzadeh, 2005
Sabinyl acetate (34.2%)	Armenia	Herbal products purchased from retail pharmacies in European countries, lab-distilled	Orav et al., 2006
Sabinyl acetate (0%–18.6%)	Belgium		Orav et al., 2006
α-Thujone (0.1%–64.6%), myrcene (0.2%–29.9%), sabinyl acetate (0.2%–70.5%)	Estonia		Orav et al., 2006
Sabinyl acetate (0%–84.5%)	France		Orav et al., 2006
α-Thujone (38.7%)	Greece		Orav et al., 2006
Sabinyl acetate (23.6%)	Latvia		Orav et al., 2006
Sabinyl acetate (13.7%)	Lithuania		Orav et al., 2006
Epoxyocimene (23.1%–56.6%), β-thujone (0%–40.6%), sabinyl acetate (0%–11.5%)	Italy		Orav et al., 2006
Myrcene (38.9%)	Moldova		Orav et al., 2006
*cis*-Epoxyocimene (0%–22.1%)	Russia		Orav et al., 2006
Caryophyllene oxide (25.3%), *p*-cymene (16.8%), 1,8-cineole (8.9%), and (*Z*)-lanceol acetate (7.3%)	Greece	Aerial parts	Basta et al., 2007
β-Pinene (23.8%), β-thujone (18.6%)	Iran	Aerial parts	Rezaeinodehi and Khangholi, 2008
Myrcene (10.8%), α-thujone (10.1%), *trans*-sabinyl acetate (26.4%)	Canada	Aerial parts	Lopes-Lutz et al., 2008
Sabinyl acetate (8.8%–39.2%), β-thujone (0%–30.7%), α-thujone (0%–23.5%)	Lithuania	Aerial parts	Judzentiene et al., 2009
Chamazulene (25.30%–39.93%), α-thujone (39.69%), *cis*-sabinene hydrate (5.32%–21.78%)	Morocco	Leaves	Derwich et al., 2009
Chamazulene (28.6%), spathulenol (7.4%)	Turkiye	Aerial parts	Bagci et al., 2010
*trans*-Sabinyl acetate (21.8%–51.3%), β-thujone (0.3%–32.2%), α-thujone (0.1%–12.5%), β-pinene (2.5%–12.9%), sabinene (0.5%–10.9%)	Lithuania	Aerial parts	Judzentiene and Budiene, 2010
Myrcene (8.6%–22.7%), *cis*-chrysanthenyl acetate (7.7%–17.9%)	Tajikistan	Aerial parts	Sharopov et al., 2012
1,8-Cineole (36.46%), camphor (10.2%), borneol (25.99%)	Iran	Leaves	Taherkhani et al., 2013
Sabinene (24.49%), sabinyl acetate (13.64%), α-phellandrene (10.29%)	Serbia	Aerial parts	Mihajilov-Krstev et al., 2014
Camphor (24.81%), chamazulene (13.71%)	Tunisia	Aerial parts	Dhen et al., 2014
*cis*-β-Epoxyocimene (49.3%–71.5%), (*Z*)-chrysanthenyl acetate (7.6%–18.0%)	Spain	Aerial parts	Llorens-Molina and Vacas, 2015
(*Z*)-Epoxyocimene (23.4%–39.0%), *cis*-chrysanthenol (16.0%–26.6%), (5*Z*)-2,6-dimethylocta-5,7-diene-2,3-diol (3.6%–25.9%)	Spain	Aerial parts	Julio et al., 2015
β-Thujone (6.72%–22.09%)	Tunisia	Aerial parts	Msaada et al., 2015
Camphor (12.96%–50.37%), chamazulene (5.98%–42.96%), bornyl acetate (0.2%–15.50%), α-thujone (0%–15.08%)	Tunisia	Leaves	Riahi et al., 2015
β-Thujone (0%–83.5%), intermedeol (0%–36.7%), myrcene (0%–36.4%), β-caryophyllene (0.6%–24.8%), caryophyllene oxide (0.4%–23.15%)	Hungary	Leaves	Llorens-Molina et al., 2016
α-Fenchene (1.0%–42.8%), bornyl acetate (1.7%–26.6%), geranyl isobutanoate (0.7%–22.8%), neryl isovalerate (0.5%–19.4%), myrcene (0.8%–18.0%),	Hungary	Roots	Llorens-Molina et al., 2016
*cis*-β-Epoxyocimene (59.9%–83.5%), (*Z*)-chrysanthenyl acetate (0%–25.8%)	Spain	Leaves	Llorens-Molina et al., 2016
Bornyl acetate (14.0%–55.2%), β-terpinyl acetate (0.1%–33.9%), neryl isovalerate (3.0%–24.7%), α-fenchene (0.1%–12.4%), neryl isobutanoate (0.1%–11.3%)	Spain	Roots	Llorens-Molina et al., 2016
α-Thujone (1.2%–51.7%), chamazulene (1.7%–9.7%), selin-11-en-4α-ol (1.1%–6.5%), β-caryophyllene (1.0%–6.5%)	Belgium	Leaves	Nguyen et al., 2018a
*cis*-Epoxyocimene (34.5%–65.1%), (*Z*)-*iso*-citral (10.4%–48.5%), sabinene (1.2%–38.1%), β-myrcene (3.1%–26.7%), α-phellandrene (1.4%–14.5%), (*Z*)-nuciferol isobutyrate (1.0%–20.1%), selin-11-en-4α-ol (1.3-15.4%), (*E*)-nuciferol isobutyrate (13.2%)	England	Herbal products purchased from retail shop, lab-distilled	Nguyen et al., 2018a
β-Thujone (2.1%–85.2%), β-myrcene (3.6%–68.1%), *trans*-sabinyl acetate (1.2%–94.5%), selin-11-en-4α-ol (50.8%)	Germany	Leaves	Nguyen et al., 2018a
*trans*-Sabinyl acetate (87.6%), β-myrcene (38.3%), (*E*)-nuciferol isobutyrate (33.2%), sabinene (31.1%), (*Z*)-nuciferol isobutyrate (22.4%), α-phellandrene (15.0%)	Germany	Herbal products purchased from retail shop, lab-distilled	Nguyen et al., 2018a
β-Thujone (0.3%–87.6%), *trans*-sabinyl acetate (3.4%–75.2%), selin-11-en-4α-ol (1.1%–58.0%), β-myrcene (1.1%–68.4%), linalool (1.0%–52.1%), *cis*-epoxyocimene (1.5%–45.9%), sabinene (1.2%–33.8%)	Hungary	Herbal products purchased from retail shop, lab-distilled	Nguyen et al., 2018a
β-Myrcene (1.4%–44.0%), *cis*-chrysanthenol (37.3%), selin-11-en-4α-ol (2.3%–21.8%), *E*)-nuciferol isobutyrate (3.5%–22.1%), β-caryophyllene (2.6%–19.6%)	Hungary	Herbal products purchased from retail shop, lab-distilled	Nguyen et al., 2018a
*trans*-Sabinyl acetate (20.3%–77.8%), *cis*-epoxyocimene (16.6%–29.6%), β-thujone (0.2%–26.8%), α-thujone (14.5%–24.8%)	Norway	Leaves	Nguyen et al., 2018a
*cis*-Epoxyocimene (46.9%–75.7%), *cis*-chrysanthenyl acetate (9.4%–25.9%)	Spain	Leaves	Nguyen et al., 2018a
β-Thujone (52.24%–63.41%), β-thujone (18.82%–28.15%)	Hungary	Leaves in vegetative stages	Nguyen et al., 2018b
β-Thujone (31.06%–51.99%), α-thujone (12.74%–20.68%)	Hungary	Flowers in various stages	Nguyen et al., 2018b
β-Thujone (60.41%), (*Z*)-β-epoxyocimene (15.23%)	Serbia	Leaves	Radulovic et al., 2022
β-Thujone (46.16%), (*Z*)-β-epoxyocimene (5.22%)		Flowers	Radulovic et al., 2022
β-Thujone (54.7%), chamazulene (10.05%), α-thujone (4.25%), germacrene D (4.1%)	Algeria	Aerial parts	Benmimoune et al., 2023
*cis*-Epoxyocimene (28.8%), *cis*-sabinyl acetate (38.5%)	Croatia	Aerial parts	Politeo et al., 2023
Davanone (60.2%), chamazulene (10.8%)	Saudi Arabia	Aerial parts	Aati et al., 2024
Sabinyl acetate (0%–76.02%), *cis*-chrysanthenol (0%–49.48%), 1,8-cineole (0.47%–40.64%), *cis*-epoxyocimene (0%–17.56%)	Poland	Aerial parts	Kosakowska et al., 2025
Myroxide (7.24%–44.215), davanone (35.88%), camphor (19.07%), β-myrcene (0.60%–17.78%), 3,6-dihydrochamazulene (0%–17.78%)	Tunisia	Vegetative+flowering stage	Malaspina et al., 2025
β-Thujone (18.90%), *cis*-epoxyocimene (7.88%)	Serbia	Aerial parts	Tojic et al., 2025

The genetic factors of the plant populations are characteristic core components. Different accessions of the same species can be grouped into distinct chemical clusters, suggesting that genetic traits control the biosynthetic pathways for chemical diversity. One study on Polish populations of *A. absinthium* found significant variability in both developmental and chemical traits, distinguishing several distinct chemotypes based on EO profiles. These included a pure sabinyl acetate chemotype, mixed chemotypes, and thujone chemotype (Kosakowska et al., 2025). This suggests that genetic factors are crucial in determining the chemotypes of *A. absinthium* EOs. This is demonstrated by the high degree of chemical diversity and the existence of chemical types in different populations, even when grown under similar conditions.

The phenological stage of the plant’s growth at the time of harvest is another critical characteristic. The concentration of essential oil and its chemical profile can change as the plant matures from the vegetative stage to the flowering stage. Studies have shown that the chemical composition of *A. absinthium* EOs can also vary between different parts of the plant, such as the leaves and the roots. Thujones were characterized by high concentrations in mostly leaves and flower oils compared to oils obtained from aerial parts (**Table 6**). For example, thujone and *trans*-sabinene acetate chemotypes were investigated at the different stages. The concentration of β-thujone (31.06%–63.41%) was higher than that of α-thujone (12.74%–28.15%) in both leaf and flower samples at the development stages. The concentration of *trans*-sabinyl acetate reached 70.84% in leaf oils at floral budding (Nguyen et al., 2018a). Judzentiene and Budiene (2010) determined that the concentrations of thujone isomers were 0%–0.8% in leaves, 9.3%–10.9% in flowers, and 4.5%–5.9% in fruit oils. This indicates that the maturation process influences the concentration of specific compounds. The observed chemical complexity and variety within *A. absinthium* EOs are thus a direct outcome of its geographic origin, developmental stage, harvesting time, and the specific plant organs used.

Thujones are found in various EOs in *A. absinthium* L., *Artemisia arborescens* L., *Artemisia herba-alba* Asso, *Artemisia rutifolia* Steph. Ex Spreng., *Artemisia sieberi* Besser, *Artemisia verlotiorum* Lamotte, *Artemisia vulgaris* L., *Salvia officinalis* L., *Tanacetum vulgare* L., and *Thuja occidentalis* L (Judzentiene and Buzelyte, 2006; Blagojevic et al., 2006; Mighri et al., 2010; Sharopov and Setzer, 2011; Tabari et al., 2017; Nemeth and Nguyen, 2020; Russo et al., 2020; Politeo et al., 2023). The aerial parts of *A. absinthium* are largely used in the alcoholic drink absinthe. The chronic abuse of absinthe led to a syndrome called absinthism and stimulated hallucinations after consuming it. Prolonged drinking of absinthe had caused convulsions, blindness, and mental deterioration (Nemeth and Nguyen, 2020). The toxicity of absinthe is attributed to the presence of thujones in wormwood oil. α-Thujone is more potent than β-thujone in the gamma-aminobutyric acid (GABA_A_) receptor in the central nervous system. The GABA_A_ receptor is the main inhibitory neurotransmitter receptor in the brain, responsible for calming neuronal activity. By blocking this receptor, thujone prevents inhibitory signals, leading to hyperexcitability of neurons. This can cause a range of symptoms from muscle spasm and restlessness to convulsions and seizures. A study in rats and mice showed that the intraperitoneal LD_50_ of α-thujone in mice is approximately 45 mg/kg. A “no-effect” level in rats was found to be 12.5 mg·kg^−1^·day^−1^ (Lachenmeier and Uebelacker, 2010; Pelkonen et al., 2013; Nemeth and Nguyen, 2020). In a clinical study by Dettling et al. (2004), 25 volunteers were exposed to absinthe containing high (100 mg/mL) and low (10 mg/L) concentrations of thujone. A high concentration of thujone had a negative effect on attention performance and some mood dimensions at the 30-minute examination time. Alcohol alone or with a low concentration of thujone did not result in similar effects (Dettling et al., 2004). The use of essential oils containing thujone in foods and beverages is strictly regulated. The content of thujones in wormwood EOs is up to 35% (Orav et al., 2006). In the United States, the Food and Drug Administration (FDA) and Department of the Treasury Alcohol and Tobacco Tax and Trade Bureau (TTB) consider products “thujone-free” if they contain less than 10 ppm of thujone (Department of the Treasury Alcohol and Tobacco Tax and Trade Bureau, 2007).

The market quality of essential oils, particularly those of *A. absinthium*, can vary significantly. Studies have confirmed that the essential oil composition of *A. absinthium* can vary dramatically depending on the plant’s geographical origin, genetic chemotype, phenological stage, and the specific plant tissue from which the essential oil is extracted. This supports that a wormwood essential oil from one supplier may have a high concentration of thujone. At the same time, another from a different region may be rich in epoxyocimene, chrysanthenyl acetate, sabinyl acetate, low thujone, or other compounds. Commercial *Absinthii herba* from various retailers may lead to the use of the wrong herbs, which causes not only serious health issues but also incorrect biological activity. Analytical methods would help to detect intentional and unintentional toxic chemical compounds in *A. absinthium* botanicals.

### 4.2 Bioinsecticidal survey

In preliminary short-range bioassays, *A. absinthium* samples (A_sd_ and A_1_), which contained α-thujone and α,β-thujone, were the most attractive *A. absinthium* samples (**Supplementary Figure 1**). Both α-thujone and α,β-thujone were initially as attractive as the positive control, TTO, for the first 30–45 minutes in competitive short-range bioassays. However, when α-thujone and α,β-thujone were directly compared in a competitive assay, only approximately 20% of male *C. capitata* were attracted to the treated filter paper during the peak attraction period (30–45 minutes). This limited attraction suggests that α-thujone and α,β-thujone may not be effective short-range attractants at the 10% concentration tested or that this concentration was either too low or too high. In contrast, α-thujone (<96%) as a single compound and α,β-thujone (70% of α-thujone and 10% of β-thujone; Sigma-Aldrich) as a mixture were evaluated in the short-range bioassays, whereas α-thujone was 1.17% and β-thujone was 39.87% in the total of A_sd_ oil. Further studies are needed to determine if different concentrations of these compounds can elicit greater *C. capitata* attraction in short-range bioassays. Additionally, further studies are needed using long-range attraction studies to investigate the effects of desensitization and reduced compound concentration over time on *C. capitata* attraction.

While TTO was the most attractive compound in short-range behavioral assays (Blythe et al., 2020; Tabanca et al., 2020), it performed poorly as a long-range attractant, capturing 50% fewer male *C. capitata* in field tests than trimedlure (Shelly and Epsky, 2015). TTO’s ineffectiveness at long range may be due to its complex composition, being a mixture of various monoterpenes and sesquiterpenes with molecular weights ranging from 134 to 222 g/mol, and varying attractiveness to *C. capitata* (Tabanca et al., 2020). α- and β-Thujone (152 g/mol of each) fall into this molecular weight range, and they may make them more effective long-range attractants, as their volatility and dispersion characteristics could be more consistent than a complex mixture like TTO.

Previous research by Kurtca et al. (2021) found that EO isolated from *J. foetidissima* fruit was attractive to *C. capitata* in short-range bioassays, attracting approximately 35% of male *C. capitata*. These fruit EOs contained approximately 12% α-thujone and 25% β-thujone (Kurtca et al., 2021). The higher attraction observed with the *J. foetidissima* fruit EOs compared to pure α-thujone and α,β-thujone suggests that other attractive components, in addition to the thujones, may be present in the *J. foetidissima* fruit EOs.

α-Thujone and α,β-thujone show promise as novel attractants for *C. capitata*, warranting further investigation through field tests. These compounds, when used as single components, may prove more effective as long-range attractants compared to complex natural mixtures. Additional field studies are essential to evaluate the long-range attraction of α-thujone and α,β-thujone to wild *C. capitata*. These studies should compare their attractiveness directly to trimedlure, a known and established *C. capitata* attractant, to assess their potential as new pest control agents fully.

Furthermore, both α-thujone and α,β-thujone demonstrated contact toxicity attributes when exposed to *A. suspensa* female adults. A previous study on *J. foetidissima* fruit EO found that *A. suspensa* was associated with its higher concentration of oxygenated monoterpenes, specifically identifying α- and β-thujone as major components (Kurtca et al., 2021). The median lethal dose (LD_50_) for *J. foetidissima* fruit EO was 10.46 μg/μL, while for *J. foetidissima* leaf EO, it was 22.07 μg/μL. This difference in toxicity was explicitly attributed to variations in the chemical composition between the two essential oils, including α,β-thujone. This strong correlation suggested thujone’s direct contribution to the observed toxicity. In the current study, α,β-thujone exhibited 1.5 times more toxicity than α-thujone. β-Thujone is quite promising for toxicity testing against *A. suspensa* female adults. Supporting their insecticidal property, α,β-thujone also induced 75% mortality in adult *B. dorsalis* via an ingestion toxicity test (Jaffar and Lu, 2022). A study found that thujone was topically toxic against western corn rootworms (*Diabrotica virgifera virgifera*) (Lee et al., 1997). Both thujones are known as neurotoxins, especially α-thujone, which is considered more potent than β-thujone. Their neurotoxic effect stems from their ability to block GABA receptors, which are crucial for inhibitory neurotransmission in insects. By blocking GABA receptors, thujones disrupt normal nerve function, leading to paralysis, convulsions, and ultimately death (Hold et al., 2000 and 2001). While α-thujone displayed higher toxicity, β-thujone also showed insecticidal effects and had significant behavioral impacts. For example, β-thujone was found to modify the probing behavior of the peach potato aphid (*Myzus persicae*), acting as a deterrent, limiting aphids’ settling and preventing phloem penetration (Wróblewska-Kurdyk et al., 2019). The current toxicity finding of thujone provides practical implications for fruit fly management in orchards. With bait spray application, although it had limited chances for adult pest flies to be directly sprayed, the sprayed fruits, leaves, and other parts of the tree provide EO-coated surfaces that will increase the chances of landed adult flies of being killed. Also, there was potential for EO to be incorporated into bait as a component and contribute to the attract-and-kill management of fruit flies. However, further studies will be needed to identify the toxicity of thujones against fruit flies via ingestion.

Beyond direct toxicity, the α,β-thujone+camphor mixture has shown antifeedant activity against the Colorado potato beetle larvae and adults (Lazarević et al., 2022). A report on the effectiveness of the alcoholic extract of *A. arborescens* ‘Powis Castle’ in preventing apple infestation by codling moth (*Cydia pomonella*) larvae identified α-thujone as a key deterrent (Creed et al., 2015). Thujones and thujone-rich EOs may be a potential feeding deterrence at specific concentrations against certain insect pests.

This current study provides a comprehensive analysis of the role of semiochemicals within integrated pest management (IPM) strategies for controlling the Mediterranean fruit fly (*C. capitata*) and the Caribbean fruit fly (*A. suspensa*). Identifying novel, cost-effective kairomones is a critical ongoing challenge to make large-scale mass trapping and SIT programs more efficient and sustainable, particularly for small-scale organic farming. When integrated with the SIT, kairomones enhance the efficacy of SIT by reducing wild pest densities and ensuring that the released sterile insects are competitive and successful in the field. In combination with attractive attributes to other tephritid species, managing the dose level in a trap, thujones can potentially be applied in future field studies as an attract-and-kill tool in fruit fly management practices, offering naturally and potentially resistance-mitigating alternatives to conventional synthetic pesticides.

## Data Availability

The original contributions presented in the study are included in the article/**Supplementary Material**. Further inquiries can be directed to the corresponding author.
