# Supplementary material for: Chemical variability for authenticated and commercial Artemisia absinthium L. essential oils with thujones on tephritid fruit flies: Mediterranean fruit fly Ceratitis capitata (Wiedemann) and Caribbean fruit fly Anastrepha suspensa (Loew)
Source: Front Plant Sci. 2025 Nov 20;16:1674428. doi: 10.3389/fpls.2025.1674428 (PMC12675350; doi:10.3389/fpls.2025.1674428)
Supplement: Supplementary file 1 [file DataSheet1.docx]

**Supplementary material**

**Chemical Variability for Authenticated and Commercial** **of *Artemisia absinthium* L. Essential Oils with Thujones on Tephritid fruit flies:** **Mediterranean Fruit Fly *Ceratitis capitata* (Wiedemann) and Caribbean Fruit Fly *Anastrepha suspensa* (Loew)**

**Table S1**. Macroscopic examination and essential oil yield of *Artemisia* *absinthium* samples (A_1_-A_5_ and A_sd_)

**Table S2**. Correlation coefficient values and upper and lower confidence interval values for the short range-assays

**Figure S1.** Percent attraction of sterile male *C. capitata* to tea tree oil and *Artemisia* samples (A_sd_, A_1_, A_2_, A_3_, A_4_ and A5) in small cage no-choice bioassays. Fifty flies were tested per cage (*n* = 1

**Table S1**. Macroscopic examination and essential oil yield of *Artemisia* *absinthium* samples (A_1_-A_5_ and A_sd_)*

| **Sample no** | **Selling**  **type** | **Morphological**  **examination** |  | **Yield** (mL/kg) |
| --- | --- | --- | --- | --- |
| A_sd_ | Packed | **1:** %50 flowers  **2:** %12 leaves  **3.** %17 stem particles  **4.** %21 thin branches | 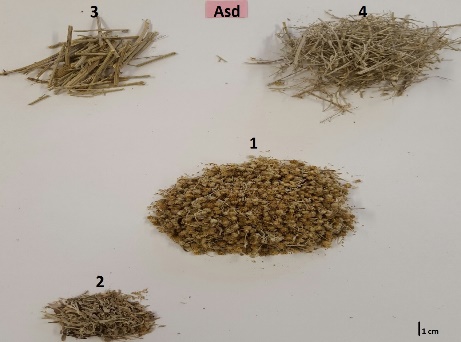 | 3.8 |
| A_1_ | Packed | **1:** %12 flowers  **2:** %55 leaf particles and powdered flower parts  **3:** %14 stem particles  **4:** %14 thin branches  **5:** %5 mixed particles | 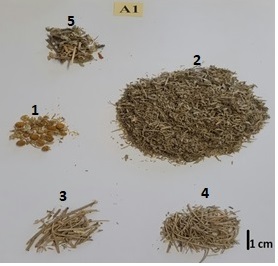 | 1.0 |
| A_2_ | Unpacked | **1:** %56 flowers  **2:** %14 leaf particles  **3:** %19 stem particles  **4:** %11 thin branches | 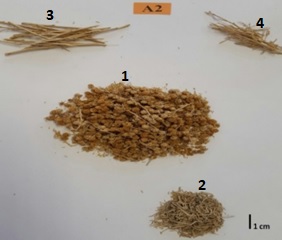 | 3.5 |
| A_3_ | Unpacked | **1:** %15 powdered flower parts  **2:** %48 leaf particles  **3:** %14 stem particles  **4:** %14 thin branches  **5:** %9 mixed particles | 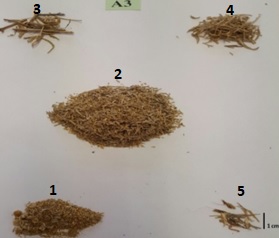 | 1.5 |
| A_4_ | Packed | **1:** %14 flowers  **2:** %31 leaves  **3:** %24 stem particles  **4:** %21 thin branches  **5:** %10 mixed particles | 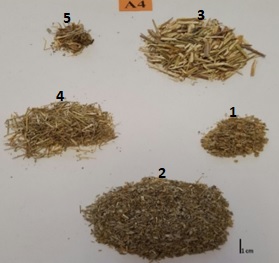 | 2.5 |
| A_5_ | Unpacked | **1:** %50 flowers  **2:** %13 leaves  **3:** %20 stem particles  **4:** % 17 thin branches | 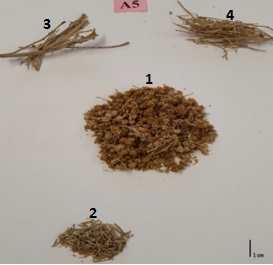 | 2.5 |

*Samples A_1_ to A_5_ were purchased from domestic markets in Turkiye. Authenticated sample of *Artemisia* *absinthium* (Asd) was obtained from Zeytinburnu Medicinal Plants Garden, Istanbul, Turkiye (see details in section 2.1 Materials).

**Table S2**. Correlation coefficient values and upper and lower confidence interval values for the short range-assays

| **TTO versus Acetone** | | | |
| --- | --- | --- | --- |
| **Minutes** | **Correlation Coefficient** | **Upper 95% C.I.** | **Lower 95% C.I.** |
| 5 | 0.41 | -7.23 | -12.76 |
| 10 | 0.1 | 12.28 | 25.71 |
| 15 | 0.14 | -19.2 | 34.47 |
| 30 | 0.34 | 27.03 | 48.98 |
| 45 | 0.05 | 35.49 | 51.85 |
| 60 | 0.39 | 29.17 | 46.49 |
| 75 | 0.31 | 26.27 | 38.73 |
| 90 | 0.14 | 19.04 | 29.29 |
| **α-Thujone versus Acetone** | | | |
| **Minutes** | **Correlation Coefficient** | **Upper 95% C.I.** | **Lower 95% C.I.** |
| 5 | 0.48 | 0.49 | 4.34 |
| 10 | 0.03 | 0.03 | 12.81 |
| 15 | 0.1 | 2.79 | 15.71 |
| 30 | 0.31 | 3.68 | -17.99 |
| 45 | 0.41 | 3.81 | 15.69 |
| 60 | 0.12 | 2.9 | 13.93 |
| 75 | 0.04 | 2.85 | 9.48 |
| 90 | 0.04 | 1.73 | 8.78 |
| **αβ-Thujone versus Acetone** | | | |
| **Minutes** | **Correlation Coefficient** | **Upper 95% C.I.** | **Lower 95% C.I.** |
| 5 | 0.75 | 0.41 | 7.42 |
| 10 | 0.59 | 3.64 | 13.68 |
| 15 | 0.25 | 4.44 | 16.57 |
| 30 | -0.18 | 5.48 | 18.02 |
| 45 | 0.06 | 5.62 | 17.21 |
| 60 | 0.41 | 4.35 | 15.32 |
| 75 | 0.43 | 5.19 | 14.98 |
| 90 | 0.15 | 5.45 | 11.71 |
| **Tea Tree Oil versus α-Thujone** | | | |
| **Minutes** | **Correlation Coefficient** | **Upper 95% C.I.** | **Lower 95% C.I.** |
| 5 | 0.09 | 4.12 | -4.63 |
| 10 | 0.61 | 5.22 | -4.89 |
| 15 | 0.54 | 4.72 | -6.72 |
| 30 | 0.49 | 10.42 | 0.58 |
| 45 | 0.63 | 11.32 | 3.17 |
| 60 | 0.47 | 10.13 | 3.03 |
| 75 | 0.69 | 10.24 | 5.25 |
| 90 | 0.54 | 9.08 | 4.07 |
| **Tea Tree Oil versus αβ-Thujone** | | | |
| **Minutes** | **Correlation Coefficient** | **Upper 95% C.I.** | **Lower 95% C.I.** |
| 5 | 0.44 | 1.98 | -2.65 |
| 10 | 0.62 | 2.45 | -6.29 |
| 15 | 0.52 | 2.78 | -7.61 |
| 30 | 0.41 | 0.67 | -10.65 |
| 45 | 0.22 | -1.35 | -11.98 |
| 60 | 0.27 | -2.94 | -12.41 |
| 75 | 0.37 | -4.42 | -11.41 |
| 90 | 0.09 | -4.29 | -11.72 |
| **α-Thujone versus αβ-Thujone** | | | |
| **Minutes** | **Correlation Coefficient** | **Upper 95% C.I.** | **Lower 95% C.I.** |
| 5 | 0.36 | 0.85 | 2.18 |
| 10 | 0.49 | 1 | 2.51 |
| 15 | 0.33 | 1.78 | 4.94 |
| 30 | 0.59 | 1.14 | 5.14 |
| 45 | 0.59 | 1.13 | -5.01 |
| 60 | 0.29 | 2.59 | 5.09 |
| 75 | 0.65 | 1.22 | -3.89 |
| 90 | 0.34 | 2.12 | 3.79 |

**Figure S1.** Percent attraction of sterile male *C. capitata* to tea tree oil and *Artemisia* samples (A_sd_, A_1_, A_2_, A_3_, _4_ and A5) in small cage no-choice bioassays. Fifty flies were tested per cage (*n* = 1)
